# Preparation and Surface Characterization of Chitosan-Based Coatings for PET Materials

**DOI:** 10.3390/molecules28052375

**Published:** 2023-03-04

**Authors:** Klaudia Szafran, Małgorzata Jurak, Robert Mroczka, Agnieszka Ewa Wiącek

**Affiliations:** 1 Department of Interfacial Phenomena, Institute of Chemical Sciences, Faculty of Chemistry, Maria Curie-Skłodowska University, 20031 Lublin, Poland; 2 Laboratory of X-ray Optics, Department of Chemistry, Institute of Biological Sciences, Faculty of Medicine, The John Paul II Catholic University of Lublin, 20708 Lublin, Poland

**Keywords:** chitosan, cyclosporine A, lauryl gallate, XPS, AFM, TOF-SIMS, contact angle, surface free energy

## Abstract

Poly(ethylene terephthalate)—PET—is one of the most frequently used polymers in biomedical applications. Due to chemical inertness, PET surface modification is necessary to gain specific properties, making the polymer biocompatible. The aim of this paper is to characterize the multi-component films containing chitosan (Ch), phospholipid 1,2-dioleoyl-*sn*-glycero-3-phosphocholine (DOPC), immunosuppressant cyclosporine A (CsA) and/or antioxidant lauryl gallate (LG) which can be utilized as a very attractive material for developing the PET coatings. Chitosan was employed owing to its antibacterial activity and also its ability to promote cell adhesion and proliferation favorable for tissue engineering and regeneration purposes. Moreover, the Ch film can be additionally modified with other substances of biological importance (DOPC, CsA and LG). The layers of varying compositions were prepared using the Langmuir—Blodgett (LB) technique on the air plasma-activated PET support. Then their nanostructure, molecular distribution, surface chemistry and wettability were determined by atomic force microscopy (AFM), time-of-flight secondary ion mass spectrometry (TOF-SIMS), X-ray photoelectron spectroscopy (XPS), contact angle (CA) measurements and the surface free energy and its components’ determination, respectively. The obtained results show clearly the dependence of the surface properties of the films on the molar ratio of components and allow for a better understanding of the coating organization and mechanisms of interactions at the molecular level both inside the films and between the films and the polar/apolar liquids imitating the environment of different properties. The organized layers of this type can be helpful in gaining control over the surface properties of the biomaterial, thus getting rid of the limitations in favor of increased biocompatibility. This is a good basis for further investigations on the correlation of the immune system response to the presence of biomaterial and its physicochemical properties.

## 1. Introduction

PET is one of the most commonly used polyesters in biomedical applications. Due to flexibility and strength PET fibers are very interesting materials in surgery and orthopedic devices applied mainly as surgical suture membranes, surgical meshes, heart valves, endovascular stent grafts, urinary and vascular catheters, scaffolds for ligament and tendon repair [1,2].

The biostability of this polymer is associated with the presence of hydrophobic aromatic groups of terephthalic acid with long crystallinity which reduces hydrolytic degradation. In spite of these beneficial features, PET fibers have some limitations in terms of the poor blood compatibility and the poor cell-adhesion properties, which are relevant for biomedical applications [3,4]. Accordingly, synthetic scaffolds can induce high incidences of postoperative infection, chronic immune response [1], compliance mismatch aside, and thrombogenicity being the major reason for artificial body substitute failure. Consequently, there is an urgent need to reduce the thrombosis process, and in parallel to strengthen the PET polymer-cell interactions and endothelial cell adhesion.

For the improvement of general and specific applications of chemically inert PET, intensive research attempts have been made for surface modification. In order to introduce different functional groups on the PET surface without changing the original bulk properties, various useful methods have been employed in many laboratories. Most of these studies are based on hydrolysis, aminolysis, graft co-polymerization, enzymatic modification, radiation, or plasma treatment [5,6].

Hydrolysis generates a mixture of –OH and $–COO^{–}$ functional groups while aminolysis introduces primary $–NH_{2}$ or secondary >NH amino groups on the PET surfaces. Both types of reactions can result in significant sample degradation due to the surface chain cleavage. Grafting is the other way, wherein the monomers can be joined onto the polymer chains through covalent bonds. This process can be activated by the chemical reaction, photoirradiation, and plasma treatment [5,6]. Plasma processing can cause the formation of the polar groups including –OH, –COOH, $–NH_{2}$, and $–SO_{4}^{-}$ on the polymer surfaces using different gases such as air, $NH_{3}$, $SO_{2}$, $CO_{2}$, and other organic compounds [6]. Plasma has been gaining a lot of popularity as a basic treatment, [7] while grafting and coating are utilized for further functionalization [6,8]. The functional groups are a key parameter in the multi-anchoring processes. The optimization strategy of biomaterials’ fabrication is focused on the multifunctional ways of immobilizing biomolecules which employ rather a mixture of biomolecules than single ones ([6] and references herein). One of the prospective approaches comprises the coverage of PET using biologically friendly substances for enhanced biocompatibility for the surrounding tissues.

To address this issue specifically, biocompatible and biodegradable natural polymers like chitosan can be used as the PET surface coatings and/or matrices for the controlled drug release [9]. Indeed, the type of the polymer is a main factor responsible for the drug release profile as well as the method applied to cover the body implant with the polymer/drug [10].

**Figure 1:**
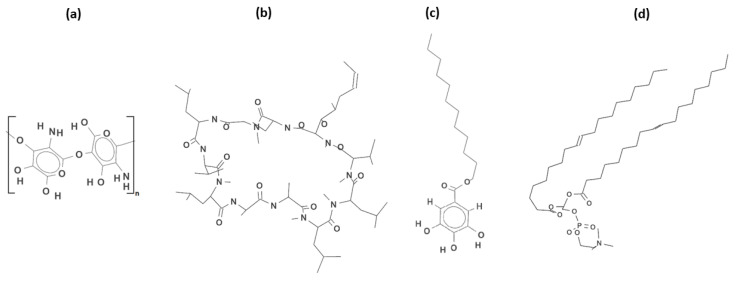
Chemical structure of chitosan monomer (Ch) (**a**), cyclosporine A (CsA) (**b**), lauryl gallate (LG) (**c**) and 1,2-dioleoyl-*sn*-glycero-3-phosphocholine (DOPC) (**d**).

Chitosan (Figure 1a), a natural polysaccharide, exhibits a unique combination of favorable biological properties such as biocompatibility, biodegradability, bioadhesiveness, non-immunogenicity, non-toxicity, wound-healing acceleration, and anti-microbial properties [10,11]. In addition, owing to its cationic character, it attracts peptides, proteins, and drugs through biological tissues and performs key functions in the endothelial cell attachment and growth [12]. Matrices or biomaterials with the chitosan coating tender many advantages in drug delivery and implant devices, including slowing the rate of degradation, the modulation of cell responses and the local release of drugs and/or growth factors from the surface of implants and tissue engineering scaffolds [10]. The polymer coating itself should be resistant to fracture and degradation, as well as firmly biocompatible and anti-thrombogenic. Moreover, a number of potential postoperative complications at the site of implantation such as recurrent narrowing, inflammatory disease or thrombosis can be minimized by the local release of drugs from the biodegradable polymers [13]. However, in spite of the great progress in artificial body implant development and interventional surgery, a lot of aspects like thrombosis and restenosis still remain to be resolved.

Undoubtedly, chitosan plays a relevant role in all processes of hemostasis. It induces the aggregation of morphotic blood components to promote the blood clotting and vasoconstriction at the injured site. The coagulant properties are strongly dependent on the interactions of positively charged chitosan with negatively charged erythrocytes, platelets, and plasma proteins. These interactions are determined mainly by the degree of deacetylation (DD) and relative molecular weight (MW) of polysaccharide [14,15]. Therefore, the appropriate selection of chitosan properties (DD, MW) in combination with the other charge-modifying components can ensure the proper balance of positive charges of chitosan-based coatings with the negative charges of blood cells and certain proteins. In consequence, it can be applied as a biocompatible structural material in the tissue reconstruction and as a matrix for the drug-released systems. This can provide the intimate contact with the tissues surrounding the biomaterial surface without causing negative side effects such as thrombosis.

Lately chitosan and its derivatives have shown perspective in the restenosis treatment. For instance, sirolimus containing chitosan-coated liposomes were found to be a novel local anti-restenosis drug carrier of sustained release behavior over conventional liposomes [16]. The stent covered by electrospinning with the chitosan/poly-cyclodextrin based nanofibers loaded with simvastatin was reported as promising for restenosis prevention [17]. Moreover, the core-shell drug encapsulated nanofibers fabricated by the co-assembly of chitosan and paclitaxel for the metal stent coating, inhibited platelet adhesion significantly, and exhibited relatively good hemocompatibility [18]. In addition, the metal stents coated with the chitosan/hyaluronic acid loading antibodies can repair vascular damages by capturing the own hematopoietic stem cells, thus contributing to their rapid natural repair, avoiding inflammation and rejection, thrombosis, and restenosis [19]. Finally, the in vitro tests with sirolimus-eluting from the chitosan-silica hybrid coating for the coronary stents evidenced great cytocompatibility and hemocompatibility, with a slight risk of in-stent restenosis and thrombogenicity [12]. As follows from the above studies, the application of chitosan-coated stents has become a new therapeutic approach for the cardiovascular disease. 

Accordingly, our attention was focused on the chitosan-based coatings for PET materials which can act as efficient matrices for the localized release of the therapeutic agent-cyclosporine A (CsA, Figure 1b). This is an immunosuppressive drug used commonly for the prevention of organ transplant rejection, in part owing to its unique ability to inhibit the activation of T cells without impairing the myeloid cell activity [20]. CsA is a cyclic undecapeptide of high lipophilicity and low permeability through the biological barriers (gastrointestinal tract, skin, and cornea) which entails inefficient administration of this poorly water–soluble drug via the oral route. Moreover, the oral formulations of CsA show high pharmacokinetic variability and low bioavailability confirmed by the unstable and incomplete absorption which is disadvantageous for efficient therapy [21,22]. In principle, the insertion of a drug on the covered implant can provide a better outcome. On the other hand, the major clinical concern is CsA-induced adverse side effects, including hepatotoxicity, nephrotoxicity, cardiotoxicity, and neural toxicity [20,23]. As the elucidation, some studies demonstrated that the CsA treatment provokes the excessive oxidative stress caused by the reactive oxygen species (ROS) and free radicals which injure chemically the biological molecules like lipids, proteins, nucleic acids, thus leading to inflammation, cell aging and death [23,24]. Although the molecular mechanism of CsA-induced toxicity is still unclear, it is obvious that antioxidants can play a beneficial role in mitigating these side effects. 

As safety is a crucial issue to consider during the novel delivery system’s development, it is essential to design new chitosan-based biocoatings for PET materials that could be used for drug incorporation, its localized release and absorption in blood. Additionally, to overcome the shortcomings faced by the CsA drug, an antioxidant can be used simultaneously. Studies on the effect of the combination of gallic acid as a strong antioxidant and cyclosporine A revealed the improvement in cardiac function [25]. The application of the gallic acid derivative, lauryl gallate (LG, Figure 1c) with the C12 hydrophobic chains, would be more interesting due to its greater antioxidant [26], membrane [27] and surface activities. The LG is required to be adsorbed at the solution/air interface and creates the prospect for employment of the Langmuir and Langmuir-Blodgett technology in the biocoating production. Both techniques provide the easy control of molecular composition, density, elasticity, and arrangement suitable for the preparation of highly ordered thin films with the molecular-level precision to be exploited in the tissue engineering [28].

Obviously, the fundamental aspect of biomaterials’ development intended for contact with living biological cells or fluids is the precise characteristics of their surface. Generally, the physicochemical properties such as topography, chemical composition, and wettability of the biomaterial used as blood-contacting devices play an important role in the activation of platelets and in the initial cell attachment determining the overall proliferation and differentiation yield of the biomaterials [29,30]. Some studies indicate that surfaces with a balanced distribution of hydrophilic and hydrophobic microdomains exhibit an optimized hemocompatibility [29,31]. Thus, the surface structure and chemistry govern a reduction in the protein adsorption and coagulation resulting in enhanced biocompatibility. 

In this paper, the Langmuir monolayer technique was utilized to fabricate the multicomponent monolayers at the chitosan solution/air interface, and to determine optimized conditions for the chitosan-based films’ transfer onto the PET substrate by means of the Langmuir-Blodgett methodology. Since chitosan by itself exhibits a poor surface activity [32], it was a component of the subphase. On the other hand, based on the amphiphilic properties of biomolecules, which are capable of self-assembling at the interface, LB technology made it possible to prepare the phospholipid 1,2-dioleoyl-*sn*-glycero-3-phosphocholine (DOPC, Figure 1d) monolayer mimicking a cell membrane and thereby facilitating the incorporation of biologically relevant molecules, i.e., immunosuppressant CsA, and antioxidant LG. Since these three compounds (DOPC, CsA, and LG) are poorly soluble in the subphase they formed stable mixed Langmuir monolayers of varying compositions at the chitosan solution/air interface which ensured an accurate control of the molecular arrangement and a homogeneous deposition onto the PET plates. Moreover, to guarantee the attachment of the molecules to the polymer plate, its surface was pre-treated with low temperature air plasma. Finally, the LB films prepared on the PET substrate were characterized in terms of wettability, topography, molecular orientation (spatial arrangement) and chemical composition by means of the combination of contact angle measurements, atomic force microscopy (AFM), time-of-flight secondary ion mass spectrometry (TOF-SIMS), and X-ray photoelectron spectroscopy (XPS).

Biomaterial surface characteristics at the molecular level is without doubt a very promising way for the development of compatible coatings with the capability of efficient drug delivery and minute investigations of biological processes at the foreign material/tissue interface.

## 2. Results and Discussion

To design biocompatible coatings for the implants used in tissue engineering there is a need to examine the physicochemical properties of the films before (Langmuir monolayers technique) and after their transfer on the solid support by means of the Langmuir-Blodgett method. Previously the studies of the interactions occurring between the molecules forming the monolayers at the air/liquid interface were carried out [33]. The thermodynamic parameters (excess area per molecule, excess and total Gibbs energy of mixing), the changes in the surface potential, and the apparent dipole moment as well as morphology at the air/AA and air/Ch interfaces were determined. All examined monolayers at the air/liquid interface were found to be in the liquid state (L) which was proven by the compression modulus values. Additionally, it was evidenced that the chitosan presence in the subphase affects the organization of the molecules forming a monomolecular film as well as the type and magnitude of the interactions between them [33]. In this research the thickness of the monolayers at the air/liquid interface (surface pressure of 10 mN m^−1^) was determined by means of the Brewster angle microscopy (BAM). Then thin films were deposited onto the air plasma-activated PET substrate by means of the Langmuir-Blodgett technique. The modified PET surfaces were characterized by the determination of the surface chemistry (XPS), the root mean square roughness ($S_{q}$) and the height profiles (AFM), the molecular arrangement (TOF-SIMS), and the surface wettability (CA).

### 2.1. Langmuir and Langmuir—Blodgett Monolayer Analysis 

In the previous paper the morphology of all monolayers was examined by means of the Brewster angle microscopy (Supplementary materials in [33]). The BAM images confirmed that the obtained monolayers are homogeneous. In the whole range of the surface pressure no domains were visible, which is in good agreement with the monolayer miscibility expressed by the negative values of excess Gibbs energy and total Gibbs energy of mixing [33]. In this paper the relative thickness (d), changes in the area per molecule as a function of time at the air/AA and air/Ch interfaces as well as the transfer ratio (TR) values are estimated and listed in Table 1.

Firstly, according to Equation (1) the relative thickness was estimated for the monolayers studied on both AA and Ch subphases [34].

(1) $$R=\frac{I_{r}}{I_{0}}={\left(\pi \frac{d}{\lambda }\right)}^{2}\frac{{\left(n_{1}^{2}-n_{2}^{2}-1+\frac{n_{2}^{2}}{n_{1}^{2}}\right)}^{2}}{1+n_{2}^{2}}$$

where $I_{0}$ means the incident intensity, $I_{r}$ is the reflected intensity, $n_{1}$ and $n_{2}$ indicate the refractive indices of the film and pure subphase, respectively; λ is the wavelength of the incident light.

For 0.1% acetic acid the monolayer thickness values are smaller than or equal to 2.1 nm. These are strictly related to the chemical structures of the examined compounds. The smallest d value is gained for the single DOPC-CsA (Table 1). This is a result of the flatness of the CsA molecule (stiff aminoacid ring, Figure 1b) which can change its conformation in relation to the polarity of the environment [35,36,37]. On the contrary, the single LG monolayer exhibits the highest d value on the Ch subphase. The molecules forming this layer can be more vertically localized by the fact that saturated hydrocarbon chains occur in their structure (Figure 1c). The presence of the unsaturated hydrocarbon chains in the DOPC structure (Figure 1d) promotes a slightly smaller relative thickness value. Owing to the *cis* double bonds, the chains can be more inclined than in the single LG monolayer. After adding LG to DOPC-CsA to form ternary (DOPC-CsA-LG) layers, the thickness values were found to be intermediated between them (Table 1). Among the mixed monolayers similar d values are gained. The addition of chitosan molecules to the acidic liquid phase slightly affects the monolayer thickness values (Table 1). Meanwhile, an increase in the thickness of the phospholipid monolayers in the presence of chitosan was shown by Cámara et al. [38] and Wydro et al. [39]. The changes in the d value are related to the Ch capability of movement from the bulk phase to the interface. Thus, the Ch can locate in the subsurface and is able to interact with the polar heads of the phospholipid [40]. 

As a result of the interactions between the chitosan and DOPC molecules, the monolayer formed at the air/liquid interface is more densely packed [33]. Similarly, greater compression modulus values were obtained for the single LG and ternary DOPC-CsA-LG monolayers [33]. Philippova et al. found that both acetylated and deacetylated units of chitosan are capable of forming hydrogen bonds [41]. Hydrogen interactions between the $-NH_{2}$ and/or $-NH_{3}$ groups and numerous amide groups of cyclosporine A may contribute to a decrease in the thickness of the CsA monolayer. Moreover, in the aqueous medium the CsA molecules can form the so-called open conformation and the intramolecular bonds cannot be formed [35]. Thus, the interactions between the Ch and CsA molecules can be promoted.

**Figure 2:**
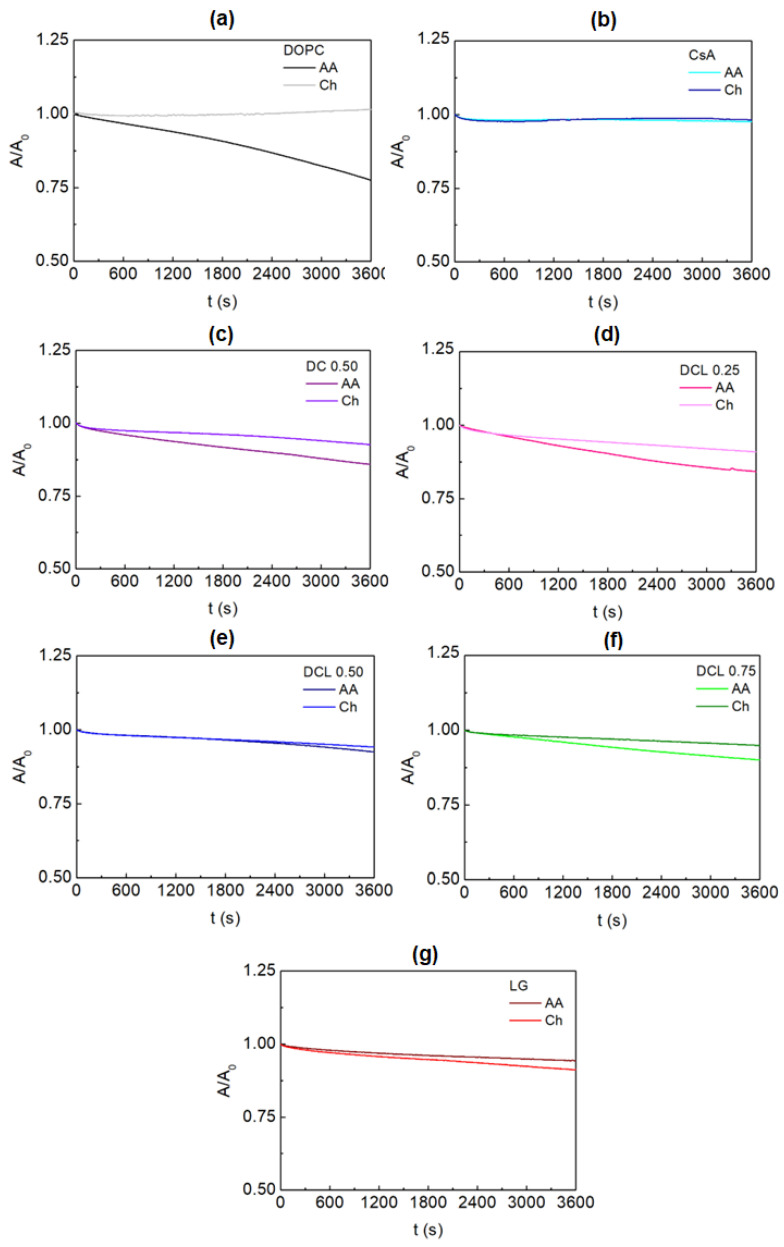
Relative molecular area ($A/A_{0}$) changes during the one-hour stabilization at 10 mN m^−1^ with the barrier speed of 5 mm min^−1^ for DOPC (**a**), CsA (**b**), DOPC-CsA 0.50 (**c**), DOPC-CsA-LG 0.25 (**d**) DOPC-CsA-LG 0.50 (**e**), DOPC-CsA-LG 0.75 (**f**), and LG (**g**) monolayers.

In the next stage of the experiments, the changes in the area per molecule in time were determined to examine the stability of the monolayer and capability of its penetration by the subphase molecules. Any loss of molecules due to desorption from the interface should be considered in the transfer ratio calculation. Figure 2 shows the relative changes in the area per molecule after the monolayer compression to the surface pressure of 10 mN m^−1^ and keeping it stable for 1 h with the constant speed of the barriers equal 5 mm min^−1^. The single DOPC monolayer at the air/AA interface is characterized by 23% reduction of the area per molecule (Figure 2a). This is an evidence that the added AA molecules can interact strongly with the phospholipid molecules promoting their desorption. Such interactions result in the monolayer molecules’ removal from the interface into the bulk liquid phase. On the other hand, the single CsA (Figure 2b) and LG (Figure 2g) films are more stable (only 2% and 6% decline, respectively). The mixed monolayers which contain the DOPC molecules exhibit the $A/A_{0}$ decrease of 8–16% (Figure 2c–f).

The presence of the Ch molecules in the subphase results in better stability of the examined monolayers (Figure 2) except for the single LG layer (Figure 2g). Surprisingly, the smallest desorption is observed for the single DOPC monolayer (1%, Figure 2a). This proves the existence of strong interactions between the phospholipid and chitosan molecules through the Lifshitz-van der Waals forces and Coulombic interactions. Therefore, it can be stated that the chitosan molecules form electrostatic complexes with the lipid and/or are accommodated in the lipid monolayer through the hydrophobic interactions [39]. 

After the chitosan addition to the subphase for the single CsA layer, the 2% decrease in the relative molecular area in time is registered (Figure 2b). This can prove that the interactions between the CsA and chitosan molecules are weaker than between the DOPC and chitosan ones. This can be a consequence of the hydrophobic nature of the CsA and Ch. However, the interactions between the molecules through the hydrogen bonds (chitosan $-NH_{2}$/$-NH_{3}^{+}$ groups and cyclosporine amide groups, Figure 1) can take place. In the case of the single LG, the interactions with chitosan occur between the hydrophobic chain (LG, Figure 1c) and the polysaccharide skeleton (Ch, Figure 1a) through the Lifshitz-van der Waals forces. Moreover, numerous hydroxyl groups of the LG can interact with the polar groups of the chitosan. For the mixed (binary and ternary) monolayers the smaller desorption occurs in the chitosan molecules’ presence (5–9% decline, Figure 2c–f), whereby the smallest one is observed for the DOPC-CsA-LG 0.50 and 0.75 (Figure 2e,f, respectively). This is related to the strong attractive interactions between the molecules forming monolayers (negative Gibbs energy of mixing, the minimum for $\chi_{LG}$ = 0.50 [33]).

In order to confirm the effectiveness of the monolayer deposition the transfer ratio was accomplished. Deposition of the monolayers was carried out directly after reaching the surface pressure of 10 mN m^−1^ by withdrawal from the liquid subphase. This is due to the fact that in the first minutes the molecules forming the monolayers’ removal to the liquid phase is the smallest (Figure 2). Based on the negative changes of the mean molecular area in time, there is a need to take into account the possible loss of the molecules in the liquid subphase in the transfer ratio determination. The corrected TR values calculated according to Equation (2) are listed in Table 1.

(2) $$TR=\frac{\Delta A_{m}}{A_{s}}$$

where $\Delta A_{m}$ denotes the decrease in the monolayer surface area on the subphase and $A_{s}$ indicates the substrate coated area. 

For almost all examined monolayers TR values are close to unity for both AA and Ch subphases. This indicates that the molecules are transferred successfully onto the air plasma-activated PET substrate and the obtained LB film can be assumed to be a monolayer. 

Among the single monolayers the greatest TR values are obtained for the single DOPC layer (TR = 1.2 and 1.3, for AA and Ch subphases, respectively). The transfer ratios are smaller than those obtained for the water subphase which is related to the lower desorption of the molecules into the liquid phase when the acetic acid and chitosan are added to the subphase [42,43]. For the single CsA layer similar TR values are obtained for all examined compositions of the subphases. After mixing the CsA and DOPC molecules forming the binary DOPC-CsA layer, smaller values of TR are obtained. This is due to the repulsion between the molecules forming the monolayer [44]. The addition of the third component-LG, changes the character of the interactions to the attraction ones. The DOPC and CsA are not matched in shape (DOPC-inverted truncated cone, CsA-ring), thus LG can be a linker between them [33,44]. Whereby, the TR values obtained during the monolayer transfer from the air/AA interface are higher than in the presence of the Ch. This is due to the larger desorption process in the absence of the chitosan molecules (Table 1). Thus, the excess molecules transfer is promoted.

### 2.2. XPS Analysis

**Figure 3:**
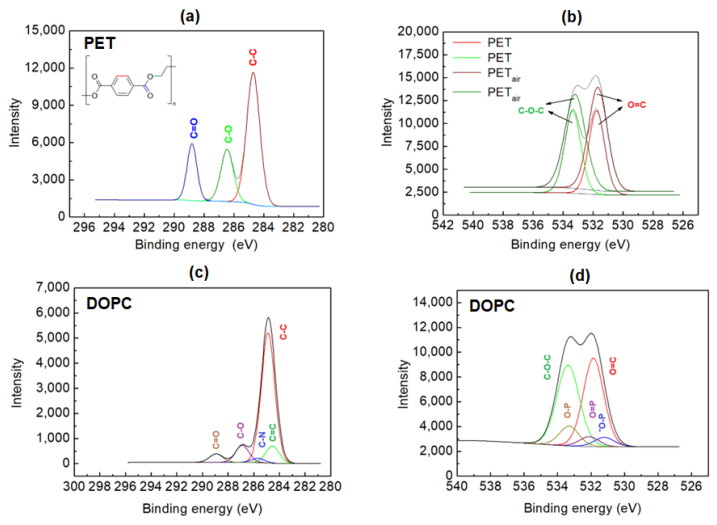
XSP spectra C 1s for unmodified PET (**a**), O 1s for unmodified and air plasma-activated PET (**b**), C 1s for PET_air_/AA/DOPC (**c**), and O 1s for PET_air_/AA/DOPC (**d**).

The XPS spectra gained for PET, PET_air_, and PET_air_/AA/DOPC are presented in Figure 3. The obtained concentration of particular elements is shown in Table 2. Both spectra and position of peaks originating from the functional groups are in agreement with those found in the literature [45,46,47,48].

Plasma treatment of the PET surface causes the increase in the nitrogen (from 1% to 2.7%) and oxygen (from 21.3% to 25.1%) concentration. The increased amount of oxygen is visible in greater peak intensities (Figure 3). Additionally, the −N= group is identified. This confirms that plasma action adds new functional groups containing these atoms onto the PET surface. The presence of the functional groups improves the PET surface hydrophilicity as well as enables the attachment of the transferred molecules by chemical bonds. In the case of O 1s XPS spectra obtained for the activated PET_air_ surface, the peaks are slightly broadened in relation to PET which can indicate that the polymer structure is changed after the plasma treatment. 

The analysis of phospholipid DOPC film on PET_air_ detects the signals of C, O, N, and P. The main peak corresponds to the aliphatic tails of the lipid [49]. The concentration of the oxygen decreases to 23.3% while that of carbon is slightly larger in comparison to PET_air_. The obtained peak areas are proportional to the number of the individual atoms within the DOPC molecule which proves the monolayer formation. The position of the combination of phosphorous with oxygen was stated according to Wagstaffe et al. [50]. The O 1s band contains contribution of several bonds such as C−O, C=O, P−O and P=O thus the deconvolution is difficult and can be encumbered with an error. Moreover, the obtained peaks’ positions are similar to those described by Panajotović et al. [49] for very similar phospholipid DPPC that possesses all saturated hydrocarbon chains. The introduction of the Ch molecules is related to the presence of the nitrogen atoms, which is not observed for the PET_air_/AA/DOPC surface. Furthermore, the Ch film resulted in a decrease in oxygen and nitrogen concentration with respect to PET_air_. This is indicative of the formation of new bonds between the activated PET surface and the Ch molecules. The analysis of mixed monolayers is very difficult due to overlapping of many peaks. A more thorough analysis was performed using mass spectrometry and is described in Section 2.4. TOF-SIMS demonstrates a significantly better detection limit than XPS and provides certain identification of monolayer species. 

### 2.3. AFM Analysis

The AFM technique was used to characterize the topography of the low temperature air plasma-modified PET surfaces with deposited chitosan film and/or Langmuir monolayers. For this purpose, the roughness parameter Root Mean Square, $S_{q}$, was determined according to Equation (3).

(3) $$S_{q}=\sqrt{\frac{1}{MN}\overset{M-1}{\underset{k=0}{\sum }}\overset{N-1}{\underset{l=0}{\sum }}{\left[z\left(x_{k},y_{l}\right)\right]}^{2}}$$

where x and y are the coordinates, z is the perpendicular deviation from the ideally smooth surface, M is the number of points in the x direction and N is the number of points in the y direction.

**Figure 4:**
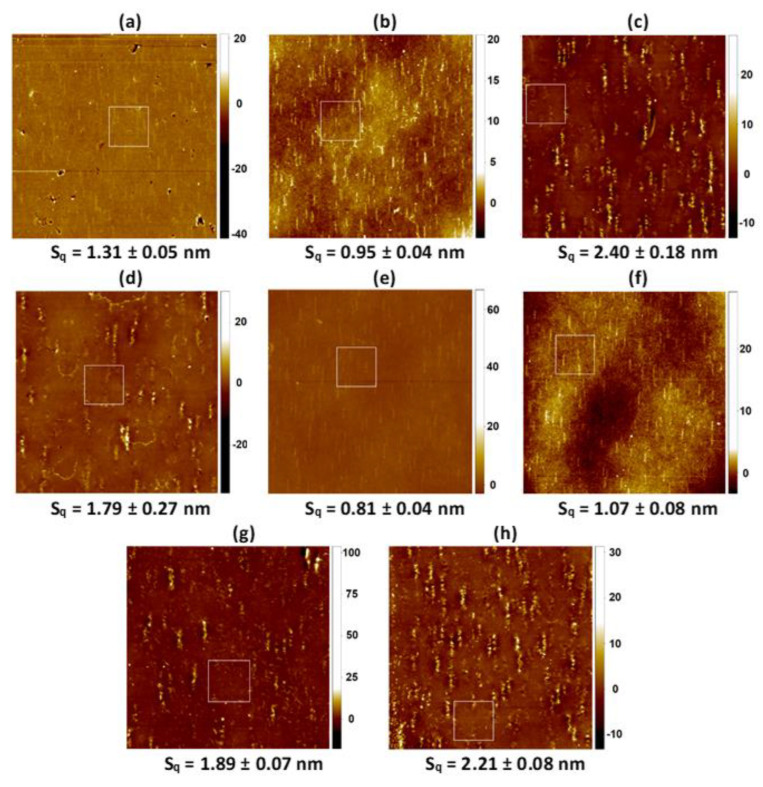
AFM micrographs for the 20 × 20 μm^2^ scanned areas: PET_air_/AA (**a**), PET_air_/AA/DOPC (**b**), PET_air_/AA/CsA (**c**), PET_air_/AA/DOPC-CsA 0.50 (**d**), PET_air_/AA/DOPC-CsA-LG 0.25 (**e**), PET_air_/AA/DOPC-CsA-LG 0.50 (**f**), PET_air_/AA/DOPC-CsA-LG 0.75 (**g**), PET_air_/AA/LG (**h**). Z scale: nm.

**Figure 5:**
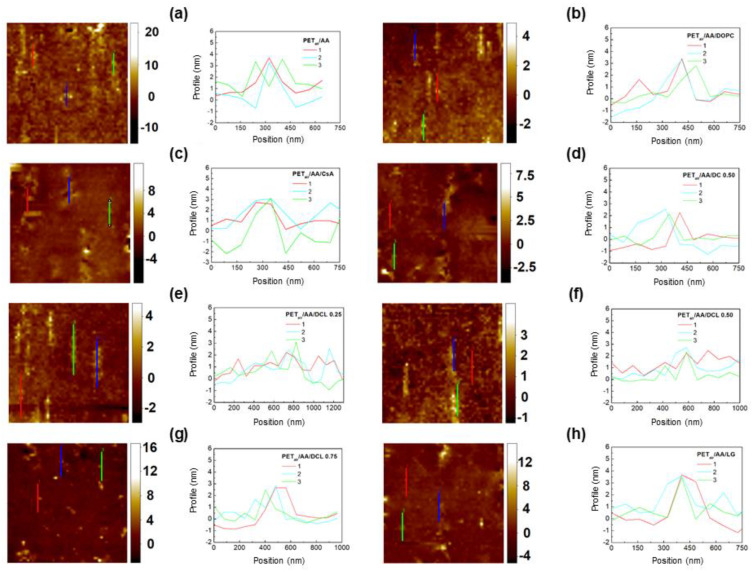
AFM zoomed micrographs and profiles obtained for: PET_air_/AA (**a**), PET_air_/AA/DOPC (**b**), PET_air_/AA/CsA (**c**), PET_air_/AA/DOPC-CsA 0.50 (**d**), PET_air_/AA/DOPC-CsA-LG 0.25 (**e**), PET_air_/AA/DOPC-CsA-LG 0.50 (**f**), PET_air_/AA/DOPC-CsA-LG 0.75 (**g**), PET_air_/AA/LG (**h**). Z scale: nm.

**Figure 6:**
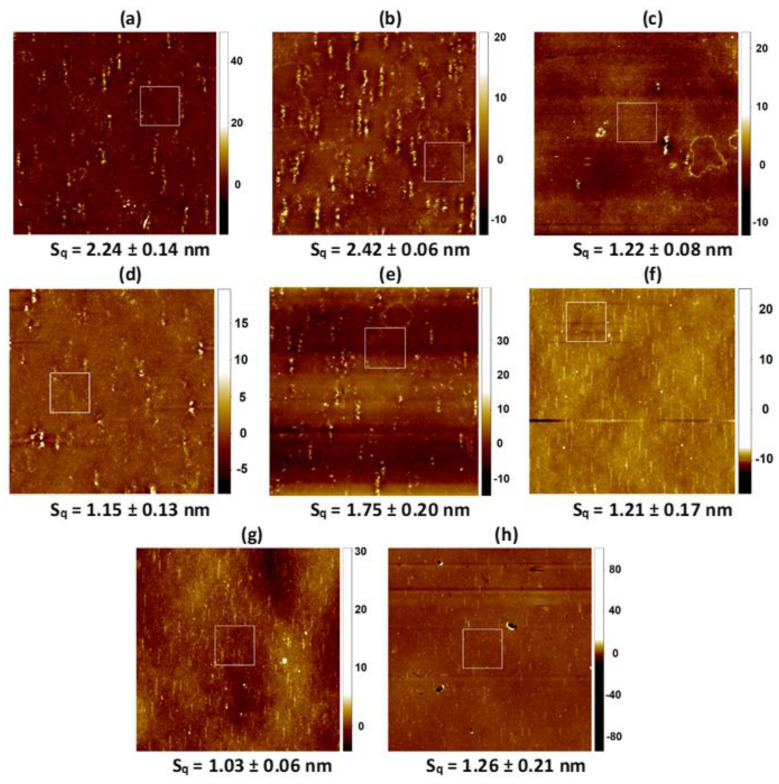
AFM micrographs for the 20 × 20 μm^2^ scanned areas: PET_air_/Ch (**a**), PET_air_/Ch/DOPC (**b**), PET_air_/Ch/CsA (**c**), PET_air_/Ch/DOPC-CsA 0.50 (**d**), PET_air_/Ch/DOPC-CsA-LG 0.25 (**e**), PET_air_/Ch/DOPC-CsA-LG 0.50 (**f**), PET_air_/Ch/DOPC-CsA-LG 0.75 (**g**), PET_air_/Ch/LG (**h**). Z scale: nm.

**Figure 7:**
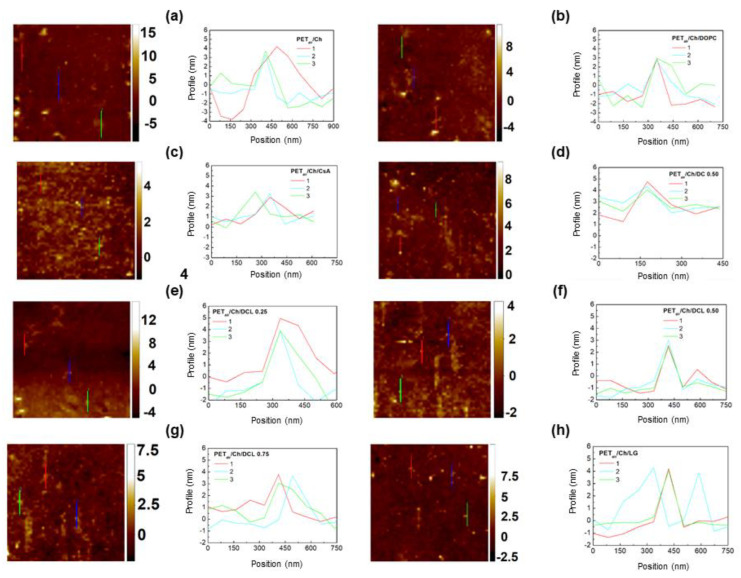
AFM zoomed micrographs and profiles obtained for: PET_air_/Ch (**a**), PET_air_/Ch/DOPC (**b**), PET_air_/Ch/CsA (**c**), PET_air_/Ch/DOPC-CsA 0.50 (**d**), PET_air_/Ch/DOPC-CsA-LG 0.25 (**e**), PET_air_/Ch/DOPC-CsA-LG 0.50 (**f**), PET_air_/Ch/DOPC-CsA-LG 0.75 (**g**), PET_air_/Ch/LG (**h**). Z scale: nm.

Figure 4, Figure 5, Figure 6 and Figure 7 show the AFM micrographs. Figure 4 and Figure 6 present the micrographs with the scanned area of 20 × 20 μm^2^ and with the marked zoomed area, while Figure 5 and Figure 7 the determined profiles.

As it was previously reported, the unmodified PET surface is smooth ($S_{q}$ ≈ 2 nm), while after the plasma modification the roughness increases to a great extent ($S_{q}$ ≈ 4 nm) [42]. The greater roughness results from the addition of the new function groups containing oxygen, nitrogen, and carbon (−OH, C−O, O=C−O, C=O, N−CO−N). Owing to them, the process of the monolayers’ transference is more effective which was confirmed by the XPS measurements (Section 2.2) as well as by the other authors [45,51,52]. Deposition of the Langmuir monolayers and/or the chitosan film results in a smoother surface (smaller $S_{q}$ parameter, Table 1, Figure 4 and Figure 6). Nevertheless, some domains are visible in the AFM micrographs, although the Brewster angle microscopy did not expose them on the microscale [33]. It proves that the molecules forming monolayers undergo reorganization during the deposition process from the liquid phase (AA, Ch) to the plasma-modified PET surface (solid support).

Of the single layers, the DOPC monolayer transfer from the AA subphase results in the biggest PET surface smoothing (Figure 2b). This finding is in good agreement with the surface roughness obtained after the deposition of the single DOPC layer from the air/water interface [42] indicating that acetic acid has no effect on the evolution of PET_air_/DOPC surface asperity. However, this time vertical domains parallel to the PET_air_ withdrawal direction with the height ca. 3 nm and the width 247 nm are visible (Figure 5b). This can be due to the change in the transfer ratio value greater than 1 which suggests that more than one monolayer is deposited onto the PET_air_ substrate (TR = 1.2, Table 1). This is probably related to the changes in the interactions between the molecules at the air/AA interface and after the film transfer onto the PET_air_ substrate. These interactions are a key point in the packing and arrangement of the thin layer onto the solid support. 

More inhomogeneous and rough surfaces are gained after the deposition of the single CsA and LG layers (Figure 4c,h, respectively). The height of the protrusions for the single CsA is in the range from ca. 2.5 nm to even 5 nm (Figure 5c) while the width is similar to that of the protrusions on the unmodified PET (380 nm) [42]. This suggests that the CsA molecules are densely packed around the protrusions similarly to those observed previously for the CsA film transferred from the water subphase [42]. This is a result of the largest stiffness of CsA among the single monolayers at the surface pressure of 10 mN m^−1^ (compression modulus equal 51.1 mN m^−1^, [33]). The TR value is equal to 1 (Table 1) which confirms the single layer deposition onto the PET_air_ substrate.

After deposition of the binary DOPC-CsA film onto the polymer substrate, a smoother surface is obtained with respect to the PET_air_/AA/CsA ($S_{q}$ = 1.79 nm, Figure 4d). The intermediate roughness between the DOPC and CsA films arises from the smaller packing of the mixed monolayer in comparison to the single CsA layer [33]. The height of the protrusions reaches 2.6 nm (Figure 5d) which is very close to those of the single DOPC and CsA layers. Similarly, the TR value is approximated to 1 (TR = 1.1).

The addition of the third component-LG, causes the smoothing of the examined surfaces (Table 1). In the group of the ternary DOPC-CsA-LG monolayers, decreasing roughness is obtained after the deposition of the DOPC-CsA-LG 0.25 and 0.50 (Figure 4e,f, respectively). It is in line with the greater packing of the monolayers obtained at the air/AA interface and better miscibility expressed by the negative values of the excess and total Gibbs energy of mixing [33]. The visible protrusions for the ternary monolayer at the LG molar fraction of 0.25 are with the height 1.7 nm and the width 226 nm (Figure 4e) while at $\chi_{LG}$ = 0.50 with almost the same height but smaller width (Figure 4f). This observation can be a reason for the changes of the surface roughness parameter (Figure 2e,f). Surprisingly, the higher LG molar fraction ($\chi_{LG}$ = 0.75) does not cause the largest smoothing of the PET_air_ surface. After the deposition of the DOPC-CsA-LG 0.75, the surface roughness is similar to that of PET_air_/AA/DOPC-CsA 0.50 (Figure 2g,e, respectively). The reason for that can be found in the smaller packing of the monolayer at the air/AA interface ($C_{s}^{-1}$ = 41.7 mN m^−1^) than for the binary DOPC-CsA layer ($C_{s}^{-1}$= 44 mN m^−1^) [33]. Moreover, the attraction interactions between the DOPC-CsA-LG molecules are weaker for the LG molar fraction of 0.75 than for 0.25 and 0.50.

After the single LG monolayer deposition, the biggest roughness of the coated PET surface is obtained if the single layers are taken into account (Figure 4h). The PET_air_/AA/LG surface is characterized by the protrusions with the height of 3.3 nm and width of 195 nm (Figure 4h). This result is contrary to the surface roughness gained after the LG film transfer from the air/water interface [42] despite the greater packing of the monolayer [33,44]. On the other hand, the thickness of the single LG layer at the air/AA interface is smaller than at the air/water interface [42]. Thus, it can be said that the acetic acid molecules influence the tilt and/or organization of the molecules forming the monolayer. Moreover, due the mismatch in the LG structure (large polar head and hydrocarbon tail, Figure 1c) the LG layer cannot form a dense LB film and as a result the molecular ion is difficult for detection in the TOF-SIMS spectra (see Section 2.4).

The adsorption of the chitosan film onto the air plasma-activated PET (PET_air_) surface is confirmed by the smaller $S_{q}$ parameter value ($S_{q}$ = 2.24 nm, Figure 6a). The effective adsorption is possible due to the interactions between the PET_air_ and chitosan hydroxyls as well as amino functional groups [53] and/or ester bonds [54]. In the AFM micrographs there are visible local aggregates which is a characteristic feature for many polysaccharides. 

Additionally, the deacetylation degree (DD) is a property of a key importance in the surface roughness characterization [55]. Chitosan with the DD equal to 82.3% used in this research has in its structure both acetylated and deacetylated groups whereby the latter are more numerous. As it was reported by the Thepsak et al. the strong binding of the chitosan film and plasma activated PE can take place [54]. The polar functional groups containing oxygen can interact with the hydroxyl groups of the chitosan to form ester linkages. This process is assisted in the acidic environment. Additionally, there are possible intermolecular hydrogen bonds between the functional groups on the PET_air_ and −OH and $-NH_{2}$ groups of chitosan [54]. For this reason, strong binding of the chitosan film to the PET_air_ surface can be obtained. 

The surface roughness of the DOPC film deposited from the chitosan subphase onto the PET_air_ substrate (PET_air_/Ch/DOPC) is larger (Figure 6b) in comparison to PET_air_/AA/DOPC (Figure 4b). It can be stated that the presence of the Ch film impacts the DOPC molecules’ organization due to the interactions between the positively charged amino groups in the acidic environment ($pK_{a}$ ≈ 6.5 [39]) and the polar choline head of the DOPC. A similar observation was reported by Cámara et al. [38]. The researchers examined the interactions between the distearoylphosphatidylglycerol (DSPG) and chitosan at the air/water interface. They correlated the increase in the packing with the fact that the chitosan molecules can locate at the interface. Additionally, on the basis of the electrostatic interactions Silva et al. proved that chitosan could form complexes with mucin and dimyristoylphosphatidic acid DMPA [56]. 

The largest smoothing is obtained for the PET_air_ substrate with the Ch film and the deposited single CsA layer (Figure 6c). The protrusions are with the height 2.3 nm and the width ca. 300 nm. These results are very similar to those obtained for the unmodified PET [42]. This can be a result of the greatest packing of the monolayer molecules obtained at the air/Ch interface for all the examined Langmuir layers [33]. The TR value is close to 1 which confirms the monomolecular layer deposition (Table 1). Additionally, the CsA monolayer shows the greatest stability over time on the chitosan subphase (Figure 2b). The presence of the Ch film results in the twice lower $S_{q}$ value for the PET_air_ substrate with the deposited Ch and binary DOPC-CsA 0.50 layers (Figure 6d) and is characterized by a smaller protrusion (height ~2.1 nm, Figure 7d) than for PET_air_/AA/DOPC. This confirms strongly that chitosan influences the interactions between the DOPC and CsA molecules. Moreover, Ch can fill free gaps developed from the structural mismatch of molecules.

The deposition of the ternary DOPC-CsA-LG monolayers alters the surface roughness of the PET_air_/Ch substrates (Figure 6e–g). For the three-component DOPC-CsA-LG $\chi_{LG}$ = 0.25 surface roughness increases with respect to the binary DOPC-CsA despite the greater packing of the molecules [33]. This is visible in the size of the protrusions (height 4.4 nm, width 255 nm). The reason for that is a specific reorganization of the film-forming molecules after their transfer to the PET_air_ substrate from the air/Ch interface. The smoothing of the surface is gained after the DOPC-CsA-LG 0.50 and 0.75 layers’ deposition from the chitosan liquid phase (Figure 6f,g). This is related to the strong attractive interactions between the molecules forming the thin films and their greater packing at the air/Ch interface in relation to the binary DOPC-CsA 0.50 layer [33]. The size of the protrusions for both ternary monolayers (DOPC-CsA-LG 0.50 and 0.75) is similar. Protrusions height and width are equal to 3.4 nm and 166 nm and 2.9 nm and 220 nm for $\chi_{LG}$ = 0.50 an 0.75, respectively.

After the single LG layer deposition from the chitosan subphase the smooth surface of PET_air_/Ch/LG is obtained ($S_{q}$ = 1.26 nm, Figure 6h). Surprisingly, there are visible height protrusions (Figure 7h, 4.1 nm). This can be related to the changes in the LG orientation induced by the interactions with chitosan through the hydrogen bonding and dispersion forces as well as smaller packing of the molecules forming monolayers [33].

Generally, the obtained TR values for all examined monolayers deposited onto the PET_air_ substrates from the chitosan subphase are close to 1 which confirms the single layer deposition. Additionally, the desorption of the molecules forming monolayers is smaller when the Ch molecules are present in the liquid subphase which indicates that monolayers are more stable. Moreover, the determined excess Gibbs energy and the total Gibbs energy of mixing proved that the obtained monolayers are more stable (stronger attraction between DOPC, CsA, LG molecules [33]) which guarantees the effective transfer of the layers.

### 2.4. TOF-SIMS Analysis

#### 2.4.1. PET and PET_air_

The TOF-SIMS measurements were made for the PET and plasma-treated PET and the characteristic peaks in the positive TOF-SIMS spectra were identified. To the best of our knowledge the peaks determined for the plasma-activated PET (Table 3) were not reported previously.

**Figure 8:**
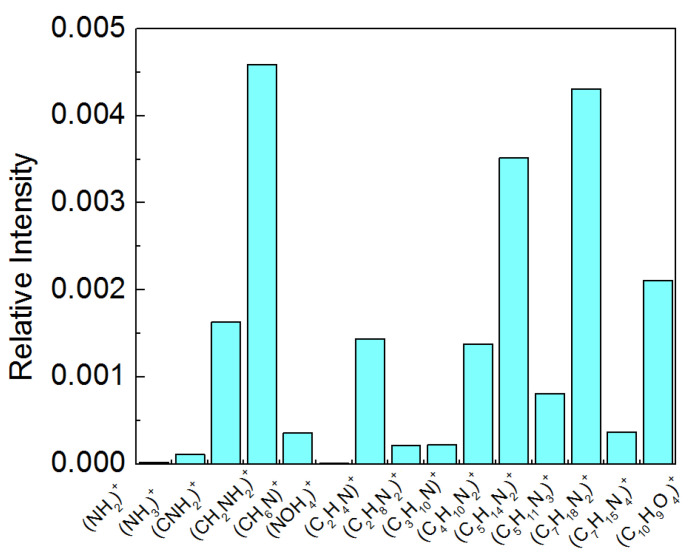
Intensity distribution of the fragments for PET_air_.

As it is shown in Table 3 there is only one common peak: ${\left(C_{10}H_{9}N_{4}\right)}^{+}$ (m/z = 193) identified in the TOF-SIMS mass spectra for PET and PET_air_. The remaining peaks were identified only for PET_air_. It can be clearly seen that during the plasma treatment the PET surface is functionalized by the $NH_{2}$ and $NH_{3}^{+}$ groups. The distribution of fragments for the PETair surface is presented in Figure 8. The intensity of the ${\left({\mathrm{NH}}_{2}\right)}^{+}$ fragment is about ten times smaller than $NH_{3}^{+}$. It means that the nitrogen atom is bound to the carbon atom via the single bond. The $NH_{3}^{+}$ fragment is yielded as follows (Equation (4)):

(4) $$NH_{2}^{•}+H^{•}\to NH_{3}^{+}$$

**Figure 9:**
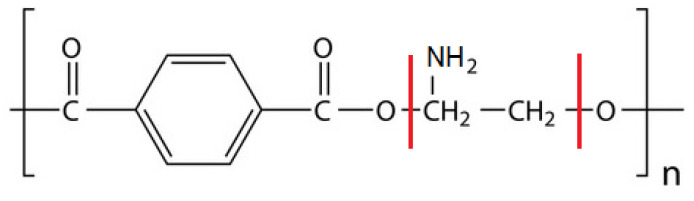
Proposed molecular structure of PET after the plasma treatment.

The existence of $NH_{2}$ rather than NH on the PET substrate is supported also by the large intensity fragment ${\left({\mathrm{CNH}}_{2}\right)}^{+}$ (Table 3). The fragment CHNH is not identified in the spectra. Moreover, it is important to distinguish that the $NH_{2}$ group is bound to the phenyl ring or hydrocarbon chain in the PET monomer. The ${\left(C_{6}H_{3}NH_{2}\right)}^{+}$ fragment is not identified while the ${\left(CHNH_{2}\right)}^{+}$ and ${\left(CH_{2}NH_{2}\right)}^{+}$ fragments demonstrate a great intensity (Figure 8). Moreover, the $NH_{2}$ group is bound to only one carbon in the hydrocarbon chain (Figure 9) since only the ${\left(C_{2}H_{4}N\right)}^{+}$ and ${\left(CH_{2}NH_{2}\right)}^{+}$ fragments are detected while the ${\left(C_{2}H_{8}N_{2}\right)}^{+}$ fragment is not observed. Furthermore, the existence of numerous peaks obtained due to the recombination of (CNH_2_)^+^ fragments with other free radical ions, suggests unambiguously that the $NH_{2}$ group resides within the hydrocarbon chain of the PET monomer unit. For example, the second prominent fragment ${\left(C_{7}H_{18}N_{2}\right)}^{+}$ (Figure 8) is yielded by cleaving the C−O bonds (Figure 9), and recombines with the other free radical fragments as follows (Equation (5)):

(5) $$CH_{2}CH_{2}NH_{2}^{•}+CH_{2}CH_{2}NH_{2}^{•}+CH_{2}CH_{2}^{•}+CH_{2}^{•}\to {\left(C_{7}H_{18}N_{2}\right)}^{+}$$

The XPS measurements were made for the selected samples. For the plasma-activated PET, the −N= functional group was identified. It plays the role of a binding agent for the deposited LB films. 

In the TOF-SIMS spectra the $NH_{2}$ group is identified. However, one should keep in mind that during the Bi^+^ bombardment plenty of highly active free hydrogen radical ions are yielded which can react easily with the nitrogen atom containing the free electron pairs. For this reason, the intensity of the ${\left({\mathrm{CNH}}_{2}\right)}^{+}$ fragment is significantly smaller than ${\left(CH_{2}NH_{2}\right)}^{+}$ since the latter fragment does not contain the free electron pairs. 

However, the identification of the −N= group by XPS is uncertain since the peak position (399.67 eV) for the PET_air_ sample corresponds to the amide and amino groups [57]. On the other hand, according to [58] after the deconvolution this energy corresponds to the −N= group. 

Taking into account the above, one can conclude that existence of the ${\left({\mathrm{NH}}_{2}\right)}^{+}$ group on the PET_air_ surface is proved by TOF-SIMS while we cannot rule out that nitrogen can exist in the highly reactive −N= form that plays a role of the binding agent for the DOPC, LG and CsA species.

The existence of DOPC is determined by the XPS spectra (Figure 3). The areas of the peaks are proportional to the number of carbon atoms in DOPC. Chitosan species are not identified due to the insufficient detection limit and the energy resolution of the XPS instrument used. It is possible that XPS equipped with a synchrotron beam will be useful for this purpose. However, we do not expect any additional information. Moreover, TOF-SIMS demonstrates a significantly better detection limit than XPS and provides certain identification of the monolayer species that in the case of XPS is very often problematic due to overlapping of many peaks. For this reason, the deconvolution can lead to improper conclusions.

#### 2.4.2. Films Deposited onto PET_air_

**Figure 10:**
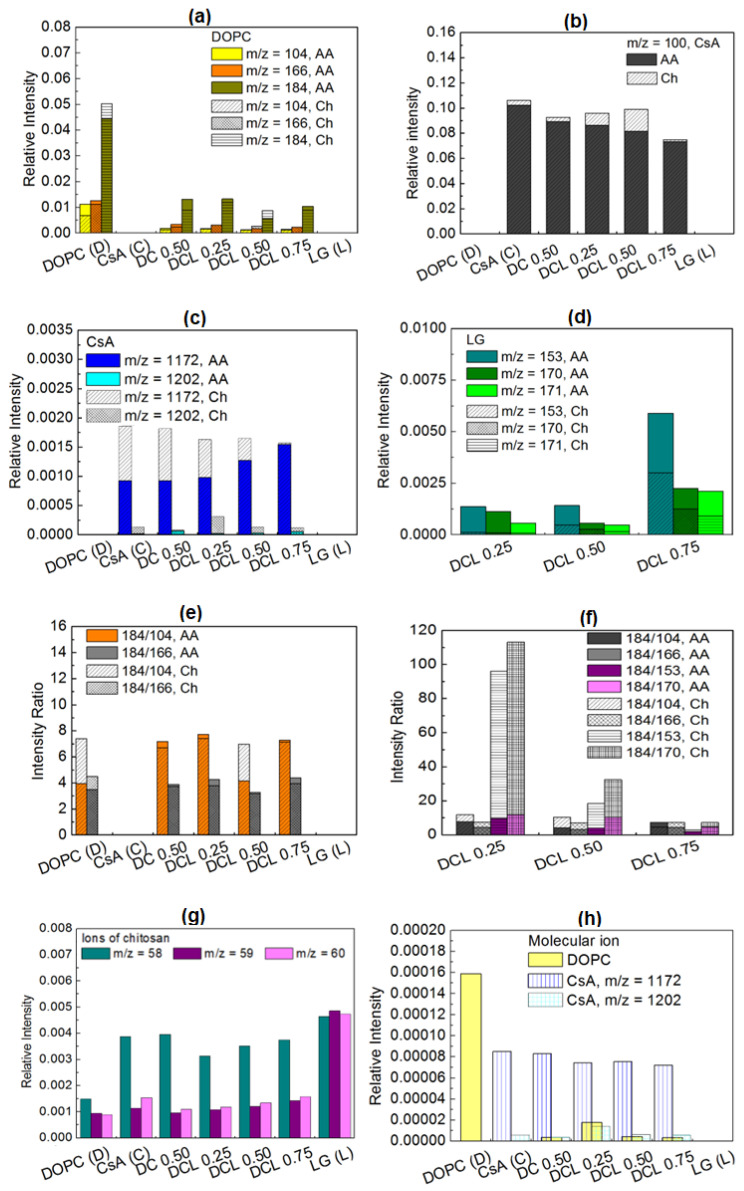
Distribution of 1,2-dioleoyl-*sn*-glycero-3-phosphocholine m/z = 104, 166, and 184 (phosphocholine) fragments (**a**), cyclosporine m/z = 100 fragment (**b**), cyclosporine m/z = 1172, 1202 fragments (**c**), lauryl gallate m/z = 153, 170, 171 fragments (**d**), intensity ratio of DOPC to DOPC fragments (**e**) intensity ratio of DOPC to DOPC and DOPC to LG fragments (**f**), distribution of the chitosan m/z = 58, 59, 60 fragments (**g**), molecular ions of the DOPC and CsA single layers (**h**) in the single, binary and ternary monolayers deposited onto PET_air_ at the air/AA and air/Ch interfaces.

The assignment m/z and identification of the most characteristic fragments of DOPC, CsA and LG for the deposited LB films on the PET_air_ substrate, prepared from acetic acid (AA) or chitosan (Ch) subphase are listed in Table 4. Furthermore, the fragments’ distribution is shown in Figure 10. There is a lack of the molecular ions of the single DOPC and LG if the LB monolayers transferred from the AA subphase are considered. The molecular ion with the mass equal to the molecular mass of the examined compound can be used as an indicator for the ordering (arrangement) of the monolayer forming molecules. The possible reason for the absence of this ion is related to the roughness and heterogeneous structure of the plasma activated PET substrate surface which was discussed in detail in our earlier paper [42]. The unsaturated bonds in the hydrocarbon chains of DOPC (Figure 1d) give rise to the steric hindrance that does not allow to form a densely packed and well-ordered layer. In the case of the LG layer, the lack of the molecular ion is in relation with the disproportion of the size of the polar head and apolar chain (Figure 1c) which provokes chain tilting to compensate for the head-tail mismatch. For this reason, monolayers occur in the liquid state with loosely packed molecules on the acetic acid subphase [33]. Assuming that the physical phase of the monolayers does not change significantly during their transfer to the solid support, the LB film molecules are prone to fragmentation due to the bismuth ion bombardment. For the same reason, the molecular ions were not determined for the single DOPC and LG layers transferred onto the PET_air_ substrate from the water subphase [42].

The intensity of the most intensive fragments of the DOPC phosphocholine head with m/z = 184 ${\left(C_{5}H_{15}{\mathrm{NPO}}_{4}\right)}^{+}$ dominates over the intensity of the fragments m/z = 166 ${\left(C_{5}H_{13}{\mathrm{PO}}_{3}N\right)}^{+}$ and m/z = 104 ${\left(C_{5}H_{14}\mathrm{NO}\right)}^{+}$ (Figure 10a). This is in good agreement with the literature data [62,63,64]. A comparable distribution of the choline head fragments was obtained for the DOPC layer without acetic acid deposited onto the PET_air_ [42] and on mica [59]. As it was previously reported, the most prominent fragments for the CsA molecule are m/z = 100 ${\left(C_{6}H_{14}N\right)}^{+}$, 1172 ${\left(C_{61}H_{107}N_{10}O_{12}\right)}^{+}$ and 1202 ${\left(C_{62}H_{112}N_{11}O_{12}\right)}^{+}$. The positive spectra fragments are consistent with the data published by the other researchers [60,65]. After mixing the DOPC and CsA molecules to gain the binary DOPC-CsA 0.50 film, the intensity of the above-mentioned DOPC fragments (m/z = 184, 166, 104) decreases c.a. 3-times (Figure 10a). On the other hand, the intensity of the most abundant fragment of CsA (m/z = 100) is reduced by about 10% for the binary film (Figure 10b). This indicates a smaller CsA amount (surface coverage) which is in line with the reduced CsA content in the DOPC-CsA monolayer at the interface (before deposition) in relation to the single CsA film. Analogous intensities of the CsA fragments were observed without the presence of acetic acid in the subphase (pure water) while the intensities of the DOPC choline head were twice smaller for the single DOPC layer and five-times smaller for the binary DOPC-CsA film [42]. 

This can suggest that a smaller amount of the DOPC molecules was transferred to the PET_air_ substrate. Due to higher ions relative intensities in the presence of the acetic acid in the liquid subphase it can be said that it stands for the more effective deposition process of the DOPC onto the PET_air_ (higher PET_air_ surface coverage with the DOPC molecules) while it does not exhibit any impact on the transfer of the CsA molecules. On the other hand, under the smaller PET_air_ surface coverage with CsA (smaller intensity of (m/z = 100, Figure 10b) intensity of the pseudomolecular ion of CsA (m/z = 1172, ${\left(C_{61}H_{107}N_{10}O_{12}\right)}^{+}$) is maintained while that of the molecular ion ${\left(C_{62}H_{112}N_{11}O_{12}\right)}^{+}$ increases in comparison to the single CsA layer (Figure 10c). This is determined by the reorientation of the CsA molecules to be more perpendicular towards the PET_air_ substrate. That latter process is more intensive than for the DOPC-CsA monolayer without acetic acid as it was presented in our earlier paper [42]. A more upright position of the CsA molecules is a natural consequence of the greater surface coverage with DOPC in the DOPC-CsA layer that reduces the sites accessible to CsA in comparison to the single CsA layer. 

The presence of the third component, lauryl gallate (LG) in the mixed monolayers transferred on the PET_air_ substrate is revealed by the occurrence of m/z = 153 ${\left(C_{7}H_{5}O_{4}\right)}^{+}$, 170 ${\left(C_{7}H_{6}O_{5}\right)}^{+}$ and 171 ${\left(C_{7}H_{7}O_{5}\right)}^{+}$ fragments (Figure 10d). The LG amount at the molar fraction of 0.25 causes a slight increase in the intensity of the CsA pseudomolecular ion and significant reduction that of the molecular ion (Figure 10c). This inconsistency can be determined by the unknown mechanism of the yield of the pseudo-molecular ion that is formed by the subtraction of two oxygen atoms from the molecular ion. Despite that fact, with the smaller amount of CsA for the ternary DOPC-CsA-LG 0.25 due to the smaller intensity of the fragment with m/z = 100 (Figure 10b), the CsA molecules have to be less tilted towards the substrate than in the case of the single CsA monolayer. This results from a slightly larger intensity of the fragment m/z = 1172 and a similar intensity of the molecular ion (Figure 10c) in comparison to the single CsA layer but under the smaller surface coverage. The changes of the geometrical orientation of CsA in the three-component DOPC-CsA-LG 0.25 film in comparison to the binary DOPC-CsA 0.50 one is difficult to estimate due to the mentioned inconsistency in changes of the pseudomolecular/molecular ion intensities. For the ternary DOPC-CsA-LG 0.50 significant reduction of the surface coverage with CsA is observed (Figure 10b,c). The intensity of the m/z = 100 ${\left(C_{6}H_{14}N\right)}^{+}$ decreases (Figure 10b) while the intensity of the pseudomolecular and molecular ions increases (Figure 10c). The latter behavior exhibits unambiguously a strong reorientation of the CsA molecules that take the most perpendicular orientation towards the PET_air_ substrate for the DOPC-CsA-LG 0.75 layer (the greatest intensity of the molecular ions, Figure 10c).

Based on the relative intensity the surface coverage with DOPC demonstrates a minimum for the ternary DOPC-CsA-LG 0.50 monolayer while for DOPC-CsA-LG 0.75 it is slightly smaller than for DOPC-CsA-LG 0.25 (Figure 10a). Similarly, an unexpected distribution of intensity of the most characteristic fragments of LG (Figure 10d) is observed. For the DOPC-CsA-LG 0.50 film the intensity of the most abundant fragment (m/z = 153) remains unchanged while that of the fragments with m/z = 170 and 171 is even reduced (with respect to the DOPC-CsA-LG 0.25). However, since the fragment with m/z = 153 is produced by a simple cleavage of the bond C−O [59] it illustrates more appropriately the surface coverage with LG than the fragments m/z = 170 and 171 which need the additional hydrogen ions for yielding. For the greatest molar fraction of LG ($\chi_{LG}$= 0.75) in the three-component layer, a significant increase in all LG fragments’ intensity can be clearly seen (Figure 10d). 

The unexpectedly smaller amount of the DOPC and LG for the ternary DOPC-CsA-LG 0.50 layer deposited on the PET_air_ substrate can be determined only by the reduced transfer of the DOPC and LG from the AA subphase onto the solid support in relation to pure water. The reason for that behavior for the DOPC-CsA-LG 0.50 film is unknown. During the deposition of the mixed DOPC-CsA-LG monolayer without acetic acid a different behavior was observed in our previous studies [42]. 

On the other hand, the intensity of all DOPC fragments for DOPC-CsA-LG 0.75 is smaller than for DOPC-CsA-LG 0.25 that can correspond to the content of DOPC in the mixed monolayers at the air/liquid interface. This fact suggests that there are no direct interactions between the hydroxyl groups of LG and the phosphate group of DOPC that affects greatly the increase in the intensity of all DOPC fragments through the transfer of hydrogen ions as it was proved in the previous paper [59]. This conclusion is additionally supported by the distribution of the 184/104 and 184/166 intensity ratio (Figure 10e). 

In the mixed DOPC-CsA-LG monolayers only a slight increase in the 184/104 and 184/166 ratios is observed suggesting the rather weak direct interactions between the DOPC and LG molecules shown above. Similarly, for ternary DOPC-CsA-LG 0.75 there are no significant changes of the 184/104 and 184/166 ratios. Moreover, the DOPC-CsA-LG 0.50 monolayer is not a representative one due to the small surface coverage (small intensity of the DOPC choline head, Figure 10a) onto the PET_air_ surface. Therefore, the sample cannot be considered in this part of the discussion. On the other hand, a significant increase in the 184/104 intensity ratio in the DOPC-CsA 0.50 layer in comparison to the single DOPC layer is observed (Figure 10e). This indicates evidently that the interactions between the CsA and DOPC molecules determine the transfer of hydrogen ions from the CsA molecules to oxygen atom bound with the phosphorus atom next to the hydrocarbon chains in the DOPC phosphate group. In this way, it gives a rise to the yield of the ${\left(C_{5}H_{15}{\mathrm{NPO}}_{4}\right)}^{+}$ fragment (m/z = 184, Figure 10a). 

The latter observation is governed by the presence of acetic acid since without this acid the direct interactions between the hydroxyl groups of LG and the phosphate group of DOPC [59] rather than between DOPC and CsA were observed. In order to improve the biocompatibility of the surface and attractive interactions between DOPC, CsA, and LG in the deposited thin film, the Ch molecules were added to the acidic liquid phase.

The distribution of the intensity of the most characteristic fragments of chitosan is presented in Figure 10g. Similarly, the other authors reported the same mass to ratio fragment of this polysaccharide [66]. The smallest intensity of the most intensive fragment of chitosan m/z = 58 ${\left(C_{2}H_{4}\mathrm{NO}\right)}^{+}$ corresponds to the largest surface coverage with the monolayer deposited onto chitosan and vice versa, the largest intensity means the smallest surface coverage with that layer. The intensities of the identified molecular ions of the single DOPC and the molecular and pseudomolecular ions of the CsA are also presented in Figure 10a,h and c,h, respectively. The DOPC molecular ion (m/z = 786) was identified for the single DOPC monolayer. The existence of the molecular ion is determined by a densely packed, uniform DOPC monolayer and indicates a great affinity of the DOPC molecules for chitosan (Figure 10h). Such a kind of monolayer was previously identified on the mica substrate [59]. Moreover, the presence of the molecular ion of different phospholipid 1,2-dipalmitoyl-*sn*-glycero-3-phosphocholine (DPPC) on the chitosan layer deposited on the PET_air_ substrate was reported in the previous paper [53]. 

Moreover, the TOF-SIMS experiments confirmed that the obtained DOPC film is the monolayer. As it was reported in the literature [67], the molecular escape depth from the organic films (tetraglyme) was equal to 1.8 nm. We can assume that the molecular escape depth for DOPC can be maintained on the similar level. It means that if the films can organize into a bilayer or a multilayer with the thickness greater than 2–3 nm, the underlying chitosan film should not be detected. As it was shown in Figure 10g, for all chitosan-containing layers there were detected the polysaccharide fragments with a varying intensity. The most prominent chitosan fragment (m/z = 58) demonstrates the smallest intensity for the single DOPC layer. This indicates that in that case the monolayer is the most ordered, dense and molecules are oriented perpendicularly to the surface. Even for this layer, chitosan is detected due to the layer thickness smaller than the molecular escape depth. If the molecular escape depth is maintained in the range 2–3 nm from DOPC/chitosan interface as it was calculated for tetraglyme, the chitosan layer can be detected. If the DOPC molecules form a bilayer, one can expect the film thickness to be in the range of c.a. 4 nm. At these circumstances, the chitosan ions should not be detected. Similarly, due to a smaller concentration of DOPC in the remaining monolayers the chitosan intensity increases due to the free gaps around the molecular species (CsA and LG).

The other argument for the monolayer formation is the occurrence of molecular ions of DOPC and CsA in the single films. The molecular ion can be identified when an appropriately dense and ordered monolayer is developed. When the intermolecular distance between the same species (DOPC or CsA) is too long, the molecular ion is not yielded due to larger fragmentation of molecular species tilted towards the substrate. This phenomenon is well known and reported in a number of papers [42,53,59].

After mixing the DOPC with the CsA molecules (DOPC-CsA 0.50) the DOPC molecular ion disappears. A small intensity of the molecular ion is observed for the ternary DOPC-CsA-LG monolayer with a small molar fraction of LG ($\chi_{LG}$ = 0.25). The intensities’ distribution of the smaller mass fragments of the single DOPC depicted in Figure 10a corresponds to the PET_air_/Ch surface coverage with DOPC. The significantly greater surface coverage with DOPC is related to the large intensity of the molecular ion while the second largest intensity (DOPC-CsA-LG 0.25) determines a densely packed DOPC monolayer confirmed by identification of the molecular ion (Figure 10h). The latter fact points out to the positive role of the LG in developing a more uniform and ordered DOPC monolayer. It is also possible that in the presence of LG, DOPC can be transferred more preferentially than from the binary DOPC-CsA monolayer. The latter hypothesis is strongly supported by the largest intensity of the ${\left(C_{5}H_{15}NPO_{4}\right)}^{+}$ (m/z = 184) fragment among two and three component layers (Figure 10a). As a result, greater amounts of the DOPC are transferred from the DOPC-CsA-LG 0.25 layer than from the binary DOPC-CsA 0.50 despite a larger molar fraction of DOPC in the binary layer than in the ternary DOPC-CsA-LG 0.25. At a higher LG molar fraction, the amount of DOPC is reduced but it is similar for DOPC-CsA-LG 0.50 and 0.75 and it is comparable to the amount transferred for the binary DOPC-CsA 0.50. This points out that a supporting role of LG in transfer of DOPC is maintained. This phenomenon is determined by the direct interactions between the DOPC and LG molecules, and it was discussed elsewhere [53,59]. 

The mutual position of the functional groups of DOPC and LG can be estimated from the distribution of the intensity ratio 184/104 and 184/166 (Figure 10f). However, the results depicted in Figure 10f do not show significant changes in the distribution of the 184/104 and 184/166 ratios. This indicates that the LG molecules on the chitosan layer can surround and penetrate the DOPC molecules at a different location of the DOPC polar choline head. As a result, any characteristic fragment: of m/z = 184, 166 and 104 (Figure 10a) is not preferentially yielded [53]. 

The surface coverage with cyclosporine A is estimated from the intensity distribution of ${\left(C_{6}H_{14}N\right)}^{+}$ fragment (Figure 10b). The largest amount of CsA is observed for the single CsA layer. After mixing DOPC with the CsA molecules the surface coverage with CsA is reduced by about 15%. Since the corresponding molecular ion intensity (Figure 10c,h) is simultaneously reduced by about 50%, it can be assumed that the orientation of CsA is changed to more horizontal towards the PET_air_ substrate. On the other hand, the intensity of the pseudomolecular ion is only slightly smaller that can suggest maintenance of the CsA orientation. However, the exact pathway of yield of the pseudomolecular ion is unclear. It is safer to use a molecular ion for estimation of geometrical orientation of the CsA molecules. Additionally, the intensities of the molecular and pseudomolecular ions are greater in the presence of the Ch film than the AA molecules.

Introducing the LG as the third component of the monolayer (DOPC-CsA-LG) leads to a slight increase in the surface coverage with CsA and a strong increase in the molecular ion intensity (Figure 10b,c). As a result, the CsA molecules are the most packed and demonstrate the most perpendicular orientation towards the PET_air_ for the LG molar fraction of 0.25. Similar behavior is observed for the DOPC (see earlier discussion for DOPC). The latter observation shows that the LG at the small molar fraction of 0.25 plays a role of an efficient agent for the DOPC and CsA molecules binding to the chitosan layer. On the other hand, the intensity of the characteristic LG fragments is very small (Figure 10d) which can be related to the competitive adsorption of the monolayer molecules from the liquid phase to the PET_air_ substrate and/or to the change of the molecules’ orientation. Furthermore, the LG molecules can be removed from the PET substrate due to the high vacuum action. For the DOPC-CsA-LG 0.50 monolayer the amount of the CsA increases further (Figure 10b) while the CsA molecules are more tilted as the intensity of molecular ion decreases (Figure 10c). That phenomenon is possible due to the smaller amount of the DOPC molecules and in consequence, larger sites accessible to the chitosan sublayer. For the largest molar fraction of LG (DOPC-CsA-LG 0.75) the amount of CsA on the chitosan layer is significantly reduced while the orientation of molecules is changed to be a more perpendicular. The intensity of the molecular ion is similar for the ternary DOPC-CsA-LG 0.50 and 0.75 (Figure 10c). The packing of the monolayer molecules and in consequence, their reorientation is determined by a significant amount of the LG molecules identified in the deposited monolayer (Figure 10c,d).

In order to examine the influence of the monolayer composition and/or the chitosan film presence on the wettability of the modified PET substrates contact angle measurements were made. 

### 2.5. Contact Angles (CA) and Surface Free Energy 

In the next stage of the experiments, the water ($\theta^{W}$), formamide ($\theta^{F}$) and diiodomethane ($\theta^{D}$) advancing contact angles were measured onto the unmodified and modified PET substrates. The exemplary images of the contact angles for water and formamide are shown in Figure S1 and Figure S2, respectively, (Supplementary Materials). The selection of such three test liquids was made on the basis of the previously conducted experiments by Jańczuk et al. [68]. Then based on the contact angle values surface free energy and its components were calculated using the Lifshitz-van der Waals acid-base (LWAB, [69,70]) model. Based on this approach the work of adhesion ($W_{A}^{a}$) of liquid (l) to solid (s) takes the form:

(6) $$W_{A}^{a}=\gamma_{l}\left(1+\cos\theta_{A}\right)=2\sqrt{\gamma_{s}^{LW}\gamma_{l}^{LW}}+2\sqrt{\gamma_{s}^{+}\gamma_{l}^{-}}+2\sqrt{\gamma_{s}^{-}\gamma_{l}^{+}}$$

The authors of the LWAB approach defined the total surface free energy as the sum of apolar $\gamma_{s}^{LW}$ and polar $\gamma_{s}^{AB}$ interactions (Equations (7) and (8)).

(7) $$\gamma_{s}^{tot}=\gamma_{s}^{LW}+\gamma_{s}^{AB}$$

(8) $$\gamma_{s}^{AB}=2\sqrt{\gamma_{s}^{-}\gamma_{s}^{+}}$$

where $\theta_{A}$ is the average advancing contact angle, $\gamma_{s}^{tot}$ is the total surface free energy, $\gamma_{s}^{LW}$ is the Lifshitz-van der Waals component, $\gamma_{s}^{AB}$ is the Lewis acid-base component, $\gamma_{s/l}^{-}$ is the electron-donor parameter of solid or liquid, $\gamma_{s/l}^{+}$ is the electron-acceptor parameter of solid or liquid. 

To calculate the $\gamma_{s}^{tot}$ value, it is essential to solve three equations of Equation (7) with the three unknowns.

**Figure 11:**
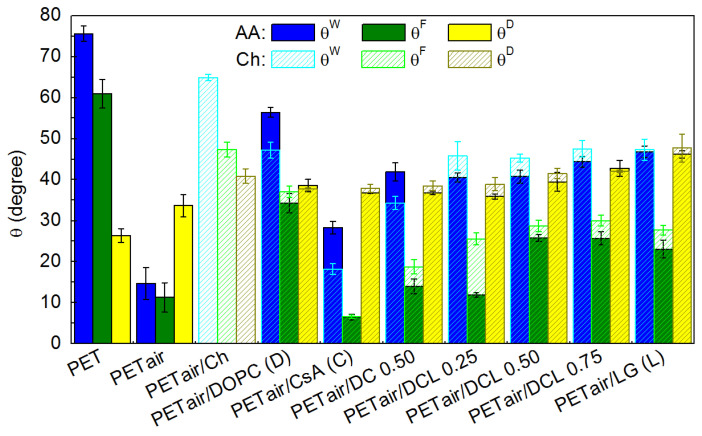
Contact angle values measured for the unmodified and modified by the LB films deposited from the air/AA and air/Ch interfaces, PET surfaces.

**Figure 12:**
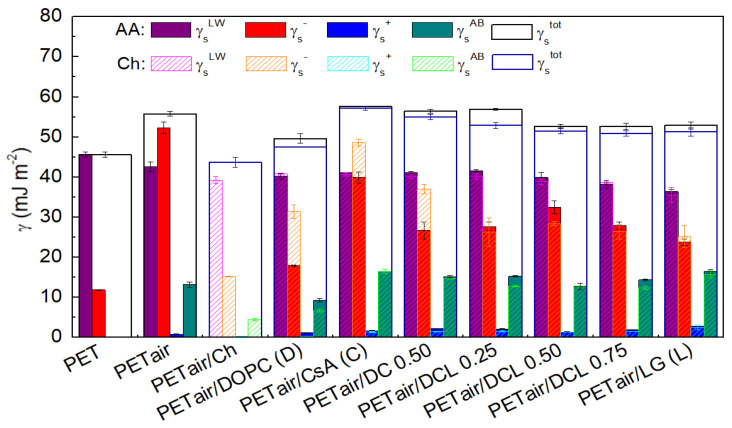
Surface free energy and its components calculated based on the LWAB approach for the unmodified and modified by the LB layers deposited from the air/AA and air/Ch interfaces onto the PET surfaces.

Figure 11 presents the measured contact angle values while Figure 12 the calculated surface free energy and its components with the standard deviations as numerical errors.

As it was previously mentioned the unmodified PET substrate is of the hydrophobic character ($\theta^{W}$ = 75.6° ± 1.9°, $\theta^{F}$ = 61.0° ± 3.5°, $\theta^{D}$ = 26.4° ± 1.7°) [42,71,72,73], which is in good agreement with the data reported by Yang et al. [7] while the plasma action changes it to be more hydrophilic (Figure 11). Such behavior confirms the addition of the new functional groups containing nitrogen, oxygen, and carbon (−OH, C−O, O=C−O, C=O, N−CO−N [45,52,54,74,75]). The reduction of the contact angle value entails larger surface free energy values (Figure 12). The unmodified PET surface is characterized by the $\gamma_{s}^{tot}$ equal to the Lifshitz-van der Waals component $\gamma_{s}^{LW}$ (45.6 mJ m^−2^, Figure 12). After the plasma activation the acid-base interactions ($\gamma_{s}^{AB}$) are found. Thus, the activated surface and the liquid molecules can interact mostly by the hydrogen bonds. The greater wettability is accompanied by the increased surface roughness of the plasma modified PET substrate [42,45]. 

The measured contact angle values for PET_air_ with the Langmuir monolayers deposited from the air/AA interface are similar to those transferred from the air/water interface [42]. This is a result of a small amount of AA in the solution whereby the dielectric constant of the AA subphase is practically equal to that of water [76]. Therefore, as for the water subphase, deposition of the monomolecular film from it onto the activated PET substrate changes the surface character to be more hydrophobic in relation to PET_air_ and more hydrophilic with respect to the unmodified PET substrate. Changes in the surface hydrophilic-hydrophobic character are correlated with the packing of the monolayer (compression modulus), morphological structure (BAM analysis) as well as interactions between the molecules forming a monolayer (excess and total Gibbs energy of mixing) [33]. The greater attractive interactions between the molecules forming the mixed monolayers, the larger packing of the layers, and thereby the modified PET surface exhibits the smaller roughness. Thus, better wettable properties are gained. 

The presence of the single (DOPC, CsA, or LG) monolayers results in the greater values of contact angles for all test liquids in comparison to the PET_air_ substrate. Among them the biggest $\theta^{W}$ is obtained for the one-component DOPC layer (56.4° ± 1.2°, Figure 11). Thus, the smaller total surface free energy is obtained where the electron-donor parameter ($\gamma_{s}^{-}$) has a significantly smaller contribution (Figure 12). Such changes can be caused by the exposition of the hydrocarbon chains of the DOPC molecules to the air whereas the polar parts are shielded from the polar liquids’ molecules. However, due to the *cis* double bonds present in the hydrocarbon chains (Figure 1d), the LB layer is loosely packed, and the chains can be inclined with respect to the PET_air_ substrate, making head groups more accessible to the polar liquid molecules. This is revealed in the $\gamma_{s}^{-}$ parameter value (17.9 mJ m^−2^). The inclination of the molecules is also confirmed by the absence of the molecular ion in the TOF-SIMS spectra (see Section 2.4). 

The most wettable surface is found for the single CsA layer, $\gamma_{s}^{tot}$ = 57.2 mJ m^−2^. In that case the greatest $\gamma_{s}^{-}$ and $\gamma_{s}^{AB}$ interactions with water and formamide occur among the LB films. Despite the largest packing which was confirmed by the compression modulus ($C_{s}^{-1}$ = 51.1 mN m^−1^) [33] and TOF-SIMS (molecular ion, see Section 2.4), the contact angles are characterized by the smallest value. This can be associated with the CsA structure (Figure 1b). The presence of the aminoacid ring causes the formation of free spaces being penetrated by the test liquids molecules making contact with the plasma modified PET substrate. 

Mixing the DOPC and CsA molecules to form the binary DOPC-CsA layer entails the decrease in the contact angle value with respect to the single DOPC layer (Figure 11), thus the increase in $\gamma_{s}^{tot}$ (56.4 mJ m^−2^). The biggest changes are observed for $\theta^{F}$, which both for the single CsA and binary DOPC-CsA acquire the smallest values ($\theta^{F}$ = 6.5° and 14.0°, respectively, Figure 11). Thus, it can be stated that the presence of the CsA molecules is mainly responsible for the acid-base interactions ($\gamma_{s}^{AB}$). Additionally, the TOF-SIMS analysis confirmed that the CsA molecules in the binary DOPC-CsA monolayer are orientated more perpendicularly towards PET_air_ than in the single CsA layer, thereby the amide groups are more accessible to the molecules of the test liquids. Moreover, the formamide molecule has in its structure the amino and carbonyl groups capable of interacting by the hydrogen bonds with the CsA molecules. 

The addition of the third component-LG to the monolayer results in a decrease in the $\gamma_{s}^{LW}$, $\gamma_{s}^{+}$, $\gamma_{s}^{AB}$ values while an increase in the $\gamma_{s}^{-}$ value (with respect to DOPC-CsA, Figure 12). This suggests that occurrence of the LG in the ternary DOPC-CsA-LG films weakens the dispersive interactions in relation to the DOPC-CsA monolayer. All the above-mentioned changes are associated with the LG structure. The hydroxyl groups bound with the aromatic ring (Figure 1c) can interact with the liquid molecules by the H-bonds. Thus, increased $\gamma_{s}^{-}$ proved that the interactions through the electron-donor groups are stronger and result in a more profound contact of the monolayers with the polar (water, formamide) test liquids. Additionally, the aromatic ring in which the π-π interactions can occur [77] also impacts the acid-base interactions. Among the ternary DOPC-CsA-LG monolayers the largest contact angles of the polar test liquids are obtained for the DOPC-CsA-LG 0.50 ($\theta^{W}$ = 40.8° ± 1.6°, $\theta^{F}$ = 25.8° ± 0.8°) and 0.75 ($\theta^{W}$ = 44.4° ± 1.3°, $\theta^{F}$ = 25.7° ± 1.6°, Figure 11). This is associated with the strong attractive interactions between the molecules forming the layer, whereby the minimum Gibbs energy for the DOPC-CsA-LG 0.50 as well as the largest packing ($C_{s}^{-1}$ = 50.1 mN m^−1^) were found [33]. As it was proved previously [42], the total surface free energy for the three-component DOPC-CsA-LG monolayers with the LG molar fractions of 0.50 and 0.75 is very similar and changes in the way of the interactions are evinced by the polar interactions ($\gamma_{s}^{-}$, $\gamma_{s}^{+}$, $\gamma_{s}^{AB}$). These differences can be ascribed to the packing of the monolayers which impacts the surface roughness (see Section 2.3). 

The single LG layer yields the largest water ($\theta^{W}$ = 46.8° ± 1.4°) and diiodomethane ($\theta^{D}$ = 46.2° ± 0.9°) contact angles among the single monolayers. Thus, the increase in the electron-acceptor ($\gamma_{s}^{+}$) and almost the double decrease in the electron-donor ($\gamma_{s}^{-}$) parameters occur which results in a slight $\gamma_{s}^{AB}$ increase, in relation to PET_air_ (Figure 12). This is correlated with the tilting of the molecules with respect to PET_air_ which makes the polar heads of LG more accessible for the interactions with the polar test liquids. Additionally, the inclination of the lauryl gallate molecules is confirmed by the mass spectrometry, where the LG molecular ion is not observed (see Section 2.4).

The air plasma-activated PET still has in the surface the aromatic rings which are a source of the π electrons. These electrons can interact readily with the positively charged $-NH_{3}^{+}$ chitosan groups. Thus, the adhesion of the chitosan film to the PET_air_ surface is strong enough to form a stable film due to the acid-base interactions between the PET functional groups and the hydroxyl and amino groups of chitosan [53]. However, the formation of the chemical bonds cannot be excluded as the XPS analysis shown. For instance, Theapsak et al. proposed the mechanism of the chitosan coating onto the polyethylene via the ester linkage formation [54]. The chitosan coating of the PET_air_ surface causes the increase in the contact angle value ($\theta^{W}$ = 64.9° ± 0.8°, $\theta^{F}$ = 47.4° ± 1.8° and $\theta^{D}$ = 40.9° ± 1.8°) in relation to PET_air_ (Figure 11). This indicates that the Ch layer leads to the weakening of the strength of the PET_air_ interactions with the test liquids. The larger contact angle values correlate with the decreased $\gamma_{s}^{tot}$ which is similar to the unmodified PET substrate (Figure 12). The basic difference is that the Ch film reveals the ability of interactions with the liquids not only by dispersive forces but also through hydrogen bonding ($\gamma_{s}^{-}$ = 15.2 mJ m^−2^ and $\gamma_{s}^{+}$ = 0.30 mJ m^−2^). 

The deposition of the examined monolayers onto the PET_air_ from the chitosan subphase substrate results in an increase in the surface polarity in relation to PET_air_/Ch (Figure 11). Among the single monolayers, the smallest contact angle of the polar test liquids and, thus the largest surface free energy ($\gamma_{s}^{tot}$ = 57.2 mJ m^−2^) are obtained for the CsA monolayer (PET_air_/Ch/CsA). Additionally, $\theta^{W}$ and $\theta^{F}$ are lower than those obtained for PET_air_/AA/CsA (Figure 11). The reason for that phenomenon are the changes in the orientation and/or conformation of the polypeptide molecules in the presence of Ch. This is proved by the mass spectrometry; the pseudomolecular and molecular ions have larger relative intensities (Figure 10c). Moreover, the intensity of the most prominent fragment (m/z = 100) points out that the surface coverage with the CsA layer is greater. Surprisingly, this behavior cannot be justified by the compression modulus value which is almost the same in the presence and absence of the chitosan layer [33]. 

The presence of the single DOPC and LG monolayers also contributes to the decrease in the water and formamide contact angle values in relation to PET_air_/Ch (Figure 11). The smaller $\theta^{D}$ is also obtained for the PET_air_/Ch/DOPC surface ($\theta^{D}$ = 37.7 ± 0.3°). Different dependence of diiodomethane contact angles is observed for the single LG monolayer. Its increase can be seen ($\theta^{D}$ = 47.7 ± 3.4°). Thus, the transfer of the single LG film changes significantly the contribution of $\gamma_{s}^{-}$, $\gamma_{s}^{+}$, and $\gamma_{s}^{AB}$, while after the single DOPC layer deposition the biggest changes in the $\gamma_{s}^{tot}$ values are related to the increase of the electron-donor parameter ($\gamma_{s}^{-}$) in comparison to the PET_air_/Ch (Figure 12). This indicates that the polar liquids molecules can have contact with the polar groups of the monolayers (−OH of LG and $-OPO_{3}^{-}$ and $-N^{+}{\left(CH_{3}\right)}_{3}-$ of DOPC). 

The binary DOPC-CsA 0.50 monolayer transfer onto the PET_air_ surface from the air/Ch interface increases the polarity of the surface in relation to the PET_air_/Ch/DOPC ($\theta^{W}$ smaller by almost 13°, and $\theta^{F}$ decreased by more than 18°, Figure 11). The smaller values of the contact angle of polar liquids result in the electron-donor parameter increase (37 mJ m^−2^, Figure 12). The addition of the LG results in the increase in the contact angles of all three test liquids (Figure 11). The higher is the LG molar fraction the more hydrophobic character of the surface is obtained (in comparison to the binary DOPC-CsA layer). On the other hand, the changes in the hydrophilic-hydrophobic character are not significant. This can be related to the similar packing of the monolayer at the air/Ch interface [33].

The wettability of the biomaterial surface is very important for assessment of the interactions of the solid with blood and surrounding tissues [29]. It was proved that the surface with the precisely designed hydrophilic-hydrophobic character can prevent from protein adsorption and coagulation [78,79]. Additionally, it was reported that the hydrophobic surfaces enhance cell affinity, while highly hydrophilic ones can prevent from cell-cell interactions [80] which are of key importance in the tissue engineering. In this experiment the deposition of the thin layers caused the increase in the contact angle value in relation to the PET_air_. Of the single monolayers the greatest wettable properties are exhibited by cyclosporine A. Due to the largest packing of this monolayer [33], the CsA molecules can be much more vertically orientated toward the PET_air_ substrate, thus its polar groups can interact readily with the liquids. In the mixed monolayers the modified PET surface has more hydrophobic character which is related to the structure of the molecules forming films. The smaller hydrophilicity is related to the strong attraction forces between the film molecules and thus their larger packing is obtained. The chitosan film presence influences the molecules orientation and wetting properties. The measured contact angles of all test liquids on the PET_air_/Ch are larger in comparison to PET_air_/AA. All ternary DOPC-CsA-LG monolayers deposited onto PET_air_/Ch seem to have desired wettable properties in the field of the tissue engineering. 

The mixed films can be used successfully to cover other substrates of medical importance, such as metals and polymers. Our previous studies showed that the DOPC-CsA-LG monolayers can be transferred effectively onto gold [43]. The obtained LB films on the gold surfaces were characterized by integrity of coating for ensuring reliability and safety of the medical devices; and uniformity and stability of the coating for avoiding localized corrosion due to local defects [43]. Additionally, the application of air plasma to the other polymers such as polyetheretherketone (PEEK) yields analogous functional groups which can ensure a similar bonding between the deposited film and the substrate surface as it was obtained in the case of PET. In this aspect, the examined films seem to be universal. The other studies presented a possibility of covering the PEEK polymer surface with CsA, phospholipid DPPC, chitosan, and very recently with bioglass [60,80,81]. The bioglass-chitosan films on the air plasma-activated PEEK were found to be stable during the 21-day incubation in the simulated body fluid.

However, in this stage of research it is difficult to determine unequivocally the LB films’ stability after their placement in the human body. The studies presented in this paper are of fundamental character and aimed at the LB layers’ characterization by means of available techniques. This research still needs to be extended before such systems can be tested in vitro and in vivo. Nevertheless, these investigations are necessary to provide unique information about the molecular organization of thin films which give the opportunity to obtain a biocompatible coating for medical devices.

## 3. Materials and Methods

### 3.1. Solution Preparation

The 1 mg mL^−1^ solutions of the single 1,2-dioleoyl-*sn*-glycero-3-phosphocholine (DOPC, ≥99%, Sigma, St. Louis, MI, USA), cyclosporine A (CsA, ≥99%, Alfa Aesar, Kandel, Germany), lauryl gallate (LG, ≥99%, Aldrich, St. Louis, MI, USA), binary DOPC-CsA 0.50 (at the 1:1 molar ratio), and ternary DOPC-CsA-LG (the LG molar fraction equal to 0.25, 0.50, 0.75) were obtained. The binary and ternary solutions were prepared by mixing proper amounts of the single ones dissolved in 4:1, *v*:*v* chloroform:methanol (chloroform 99.8%, Macron Fine Chemicals, methanol ≥99.9%, Fluka^TM^) solvents.

### 3.2. Subphase Solution Preparations

The 0.1 mg mL^−1^ chitosan (Ch) and 0.1% acetic acid (AA) solutions were used as liquid subphases. Chitosan (MW 100,000-300,000 Da, degree of deacetylation 82.3% ± 0.9, Acrōs Organics, Göteborg, Sweden) was dissolved in 0.1% acetic acid diluted from the concentrate (99.5–99.9%, Avantor Performance Materials Poland S.A. Gliwice, Poland) with water from the Milli-Q Plus 185 system (Millipore, Burlington, MA, USA) with the specific resistivity of 18.2 MΩ cm.

### 3.3. PET Substrates Modification

PET plates (20 × 20 × 3 mm^3^, Bayer Material Science, Leverkusen, Germany) were cleaned with methanol (99.8%, Avantor Performance Materials S.A. Gliwice, Poland) and deionized Milli-Q water in the ultrasonic bath (UM4, Unima, Olsztyn, Poland). Then the plates were dried in the exsiccator for 24 h at room temperature (20 ± 1°) and placed in the plasma generator chamber (Plasma type system, Diener Electronic, Ebhausen, Germany). The process of the PET surface activation with low temperature and low pressure (0.20 mbar) air plasma lasted 1 min as in our previous studies [41,69]. The power of air plasma was 460 W, and the plasma process ran with a continuous gas flow of 22 sccm. After the one-minute action, the plates were removed for further modifications. Next, the PET plates were immersed immediately in the liquid (chitosan or acetic acid) subphase.

### 3.4. Langmuir Monolayer Formation

Firstly, the Langmuir-Blodgett KSV 2000 Standard trough (KSV, Helsinki, Finland) was wiped with the acetone (99.5%, Avantor Performance Materials S.A. Gliwice, Poland) and methanol and then rinsed three times with the Milli-Q water. Then the proper volume (30–70 μL) of the solutions was placed onto the liquid subphase using the microsyringe (Hamilton-Bonaduz, Bonaduz, Switzerland). The surface tension was measured by means of the Wilhelmy plate with the 0.1 mN m^−1^ accuracy. After placing the solution on the subphase and solvent evaporation (duration of 10 min), the symmetrical compression was performed using mobile barriers with the speed of 20 mm min^−1^. At the same time, the surface pressure-area per molecule (π−A) isotherm was registered. Additionally, the possible loss of the monolayer molecules into the liquid subphase before their transfer onto the PET_air_ was examined. For this purpose, changes in the area per molecule after gaining the given surface pressure of 10 mN m^−1^ were registered with barriers moving at the speed of 5 mm min^−1^.

### 3.5. Brewster Angle Microscopy (BAM) Analysis 

The Langmuir trough coupled with the Brewster angle microscope (BAM, Accurion GmbH, Goettingen, Germany) with the lateral resolution of 2 μm was used to estimate the monolayer relative thickness at the air/liquid interface (d). At first, before placing the solution on the liquid subphase, the camera calibration was made. Thereby the plot of the grey level as a function of incidence angle was gained. The incidence angle with the smallest reflectivity constituted the minimum of the parabolic fit. After the solution spreading and the solvent evaporation, the monolayer was compressed. Simultaneously, the grey scale data were converted into the reflectivity (R) and then into the relative film thickness (d) according to Equation (1) in compliance with the single-layer optical model [34].

### 3.6. Langmuir—Blodgett (LB) Monolayer Formation

In order to deposit efficiently the monolayers onto the low temperature air plasma-activated PET substrate, the thin film at the air/liquid interface was compressed to the surface pressure of 10 mN m^−1^. After the given surface pressure had gained the PET_air_, substrate was withdrawn from the subphase through the compressed film with the barrier speed of 5 mm min^−1^. Then the PET_air_ substrate with the deposited LB layers was dried to remove water molecules and placed in the dark glass exsiccator before the next stages of the experiments (XPS, AFM, TOF-SIMS, CA).

The transfer ratio (TR) as the indicator of the efficiency of Langmuir monolayer transfer from the liquid phase to the polymer substrate and of the LB film quality was calculated according to Equation (2).

Determination of the transfer ratio value required providing the correction for the loss of the molecules during the transfer of the monolayer due to the desorption of the film material kept at the constant surface pressure.

### 3.7. X-ray Photoelectron Spectroscopy (XPS) Analysis

In order to analyze the chemical composition of the examined surfaces the X-ray photoelectron spectroscopy (XPS) studies were carried out using the multi-chamber UHV system (PREVAC). Spectra were collected by means of a hemispherical Scienta R4000 electron analyzer. The Scienta SAX-100 X-ray source (Al Kα, 1486.6 eV, 0.8 eV band) equipped with the XM 650 X-ray Monochromator (0.2 eV band) was utilized as a complementary piece of equipment. The pass energy of the analyzer was set to 200 eV for the survey spectra (with 750 meV step), and 50 eV for the regions (high resolution spectra): C 1s, O 1s and N 1s with a 50 meV step. The base pressure in the analysis chamber was 5 × 10^−9^ mbar. During the spectra collection it was not larger than 3 × 10^−8^ mbar.

### 3.8. Atomic Force Microscopy (AFM) Analysis

To examine the LB layers in the nanoscale the atomic force microscope (5600LS AFM, Aglient Technologies) was used. The tests were carried out in the non-contact mode (tip radius <7 nm, resonance frequency 330 kHz) with the resolution of 256 × 256 and the scanning area of 20 × 20 μm^2^. The measurements were made in at least three randomly selected locations on the sample. The post processing data analysis was performed using the Probe Image Processor (SPIP) v. 5.1.4 software (Image Metrology, Hørsholm, Denmark).

### 3.9. Time of Flight Secondary Ion Mass Spectrometry (TOF-SIMS) Analysis

The modified PET substrates were placed in the ultra-high vacuum (*p* < 10^−9^ mbar) chamber of the TOF-SIMS.5 instrument (ION-TOF GmbH, Münster, Germany) prior to the measurements. Subsequently, the TOF-SIMS spectra were obtained. The primary ion source of Bi^+^ was used at 30 keV and corresponded to the 1.0 pA primary beam current in the spectrometry mode where the scanning area of the secondary ions was 200 × 200 μm^2^ with 256 × 256 pixels (1 shot/pixel). All measurements were performed under the static positive conditions (dose < 1 × 10^12^ ions cm^−2^). To neutralize the charge left on the surface an electron flood gun (20 eV) and the surface potential (U = −360 V) were applied. The post-processing data analysis was conducted using SurfaceLab 6.7 software (ION-TOF) and Origin 2019 (OriginLab, Northampton, MA, USA). The spectra calibration was applied by means of the positions of $CH_{3}^{+}$, $C_{2}H_{3}^{+}$, $C_{2}H_{5}^{+}$ fragments. All fragments’ intensities were normalized to the total intensity.

### 3.10. Contact Angle Measurements (CA)

To determine the hydrophilic-hydrophobic character of the modified PET substrates the contact angle measuring was carried out (DGD ADR, GBX S.A.R.L, Romans-sur-Isére, France). For this purpose three test liquids with well-known surface tension: Milli-Q water, formamide (99.5%, Acrōs Organics, Geel, Belgium), and diiodomethane (99%, Sigma-Aldrich, St. Louis, MI, USA) were applied. For every modified PET substrate, and every test liquid, the same volume of liquid (6 μL) was placed and then using computer software Windrop++ the advancing contact angle was measured from the shape of settled droplet.

The values of water, formamide, and diiodomethane contact angles were used for further calculation of surface free energy and its components.

## 4. Conclusions

In this paper the single (DOPC, CsA, LG), binary (DOPC-CsA) and ternary (DOPC-CsA-LG) monolayers were deposited onto the air plasma-activated PET surface from the AA and Ch subphases by means of the Langmuir-Blodgett technique. Firstly, the stability of the monolayers and their relative thickness at the air/liquid interface were determined. After transferring the monolayers onto the PET_air_ the XPS, AFM, TOF-SIMS, CA measurements were made for the surface characteristics. Furthermore, the influence of the Ch film on the physicochemical properties of the modified PET substrate was examined. 

The obtained results indicated that the biggest stability of monolayers occurred in the presence of chitosan in the subphase. However, it decreased significantly with time which imposed the necessity of deposition immediately after reaching the predetermined surface pressure. The transfer ratio values estimated under these conditions were close to unity, confirming the deposition of the monomolecular films. The XPS analysis evidenced that the air plasma action added new oxygen and nitrogen containing functional groups to the PET surface. They allowed for chemical bond formation with molecules of the tested monolayers. The micrographs revealed the protrusions which can be ascribed to the molecular reorganization during the transfer process of monolayers from the subphase (AA, Ch) to the solid PET surface. The Ch film presence modulated the roughness of the examined surfaces which is promising for the hemocompatibility of the modified PET. The molecular ions identified by the TOF-SIMS analysis proved that chitosan molecules improved transfer of more packed and well-organized DOPC-CsA-LG monolayers. The water contact angle values measured for the mixed films were in the range 30–50° which can ensure the optimal hydrophilic-hydrophobic character of the biomaterials used in the tissue engineering. 

The studies allowed for the comprehensive characterization of physicochemical properties of the coatings such as composition, topography, molecular arrangement, and wettability on the solid support, useful for their potential applications in biomedicine. Based on the above findings it can be concluded that regardless of composition (DOPC-CsA-LG 0.25, 0.50, 0.75) all chitosan-containing films on PET_air_ have appropriate characteristics to be employed successfully. We hope that such organized coatings after the in vitro and in vivo tests will be used to increase the biocompatibility of medical devices.

## Figures and Tables

**Table 1:** Thickness (d) and transfer ratio (TR) values for the indicated monolayers obtained at the air/AA and air/Ch interfaces as well as the $S_{q}$ roughness parameter gained by means of the AFM for the modified PET substrates.

Subphase	0.1% AA			0.1% mg mL^−1^ Ch		
Monolayer	d (nm)	TR	$S_{q}$ (nm)	d (nm)	TR	$S_{q}$ (nm)
**DOPC**	2.0 ± 0.1	1.2 ± 0.1	1.31 ± 0.05	2.0 ± 0.1	1.3 ± 0.1	2.42 ± 0.06
**CsA**	1.9 ± 0.1	1.0 ± 0.1	2.70 ± 0.04	1.7 ± 0.1	0.9 ± 0.1	1.22 ± 0.08
**DC 0.50**	1.7 ± 0.1	1.1 ± 0.3	1.79 ± 0.28	1.6 ± 0.1	1.0 ± 0.2	1.15 ± 0.13
**DCL 0.25**	2.0 ± 0.1	1.0 ± 0.1	0.81 ± 0.27	1.8 ± 0.1	0.9 ± 0.1	1.75 ± 0.20
**DCL 0.50**	2.0 ± 0.1	1.2 ± 0.1	1.07 ± 0.08	1.9 ± 0.1	0.9 ± 0.2	1.21 ± 0.17
**DCL 0.75**	2.1 ± 0.1	1.2 ± 0.2	1.89 ± 0.07	2.3 ± 0.1	0.8 ± 0.1	1.03 ± 0.06
**LG**	2.1 ± 0.1	1.0 ± 0.1	2.21 ± 0.08	2.5 ± 0.1	0.9 ± 0.2	1.26 ± 0.21

**Table 2:** XPS data for unmodified and modified PET surfaces (conc. in at.%).

Element	PET	PET_air_	PET_air_/AA/DOPC	PET_air_/Ch	PET_air_/Ch/DOPC
C 1s	77.7	72.2	75.8	74.2	72.4
N 1s	1.0	2.7	-	0.9	3.0
O 1s	21.3	25.1	23.3	24.9	23.8
P 2p	-	-	0.8	-	0.7

**Table 3:** Peaks identified for the PET and PET_air_ surfaces in the positive TOF-SIMS mass spectra.

Assignment	m/z	Identification
${\left(NH_{2}\right)}^{+}$	16	PET_air_
${\left(NH_{3}\right)}^{+}$	17	PET_air_
${\left(CNH_{2}\right)}^{+}$	28	PET_air_
${\left(CH_{2}NN_{2}\right)}^{+}$	30	PET_air_
${\left(CH_{6}N\right)}^{+}$	32	PET_air_
${\left(NOH_{4}\right)}^{+}$	34	PET_air_
${\left(C_{2}H_{4}N\right)}^{+}$	42	PET_air_
${\left(C_{4}H_{10}N_{2}\right)}^{+}$	86	PET_air_
${\left(C_{5}H_{14}N_{2}\right)}^{+}$	102	PET_air_
${\left(C_{5}H_{11}N_{3}\right)}^{+}$	113	PET_air_
${\left(C_{7}H_{18}N_{2}\right)}^{+}$	130	PET_air_
${\left(C_{7}H_{15}N_{4}\right)}^{+}$	155	PET_air_
${\left(C_{10}H_{9}N_{4}\right)}^{+}$	193	PET, PET_air_

**Table 4:** The most characteristic positive fragments in the TOF-SIMS mass spectra obtained for all examined monolayers.

Assignment	m/z	Identification	References
${\left(C_{5}H_{14}NO\right)}^{+}$	104	DOPC	[59]
${\left(C_{5}H_{13}PO_{3}N\right)}^{+}$	166	DOPC	[59]
${\left(C_{5}H_{15}NPO_{4}\right)}^{+}$	184	DOPC	[59]
${\left(C_{7}H_{5}O_{4}\right)}^{+}$	153	LG	[59]
${\left(C_{7}H_{6}O_{5}\right)}^{+}$	170	LG	[59]
${\left(C_{7}H_{7}O_{5}\right)}^{+}$	171	LG	[59]
${\left(C_{6}H_{14}N\right)}^{+}$	100	CsA	[60]
${\left(C_{61}H_{107}N_{10}O_{12}\right)}^{+}$	1172	CsA pseudo-molecular ion	[60]
${\left(C_{62}H_{112}N_{11}O_{12}\right)}^{+}$ ${\left(CsA+H\right)}^{+}$	1202	CsA molecular ion	[60]
${\left(C_{2}H_{4}NO\right)}^{+}$	58	Chitosan	[61]
${\left(C_{2}H_{5}NO\right)}^{+}$	59	Chitosan	
${\left(C_{2}H_{6}NO\right)}^{+}$	60	Chitosan	[53]

## Data Availability

Not applicable.

## Supplementary Materials

The following supporting information can be downloaded at: https://www.mdpi.com/article/10.3390/molecules28052375/s1. Figure S1: Water contact angle images for the indicated LB films on PET_air_, Figure S2: Formamide contact angle images for the indicated LB films deposited on PET_air_.
