# Supplementary material for: Preparation and Surface Characterization of Chitosan-Based Coatings for PET Materials
Source: Molecules. 2023 Mar 4;28(5):2375. doi: 10.3390/molecules28052375 (PMC10005435; doi:10.3390/molecules28052375)
Supplement: Supplementary file 1 [file molecules-28-02375-s001.zip › molecules-2102404-supplementary.pdf]

Preparation and Surface Characterization of Chitosan-Based Coatings for PET Materials

Klaudia Szafran [1,*], Małgorzata Jurak [1], Robert Mroczka [2] and Agnieszka Ewa Wiącek [1,*]

[1] Department of Interfacial Phenomena, Institute of Chemical Sciences, Faculty of Chemistry, Maria Curie-Skłodowska University, 20031 Lublin, Poland
[2] Laboratory of X-ray Optics, Department of Chemistry, Institute of Biological Sciences, Faculty of Medicine, The John Paul II Catholic University of Lublin, 20708 Lublin, Poland
* Correspondence: klaudia.wozniak@poczta.umcs.lublin.pl (K.S.); agnieszka.wiacek@mail.umcs.pl (A.E.W.)

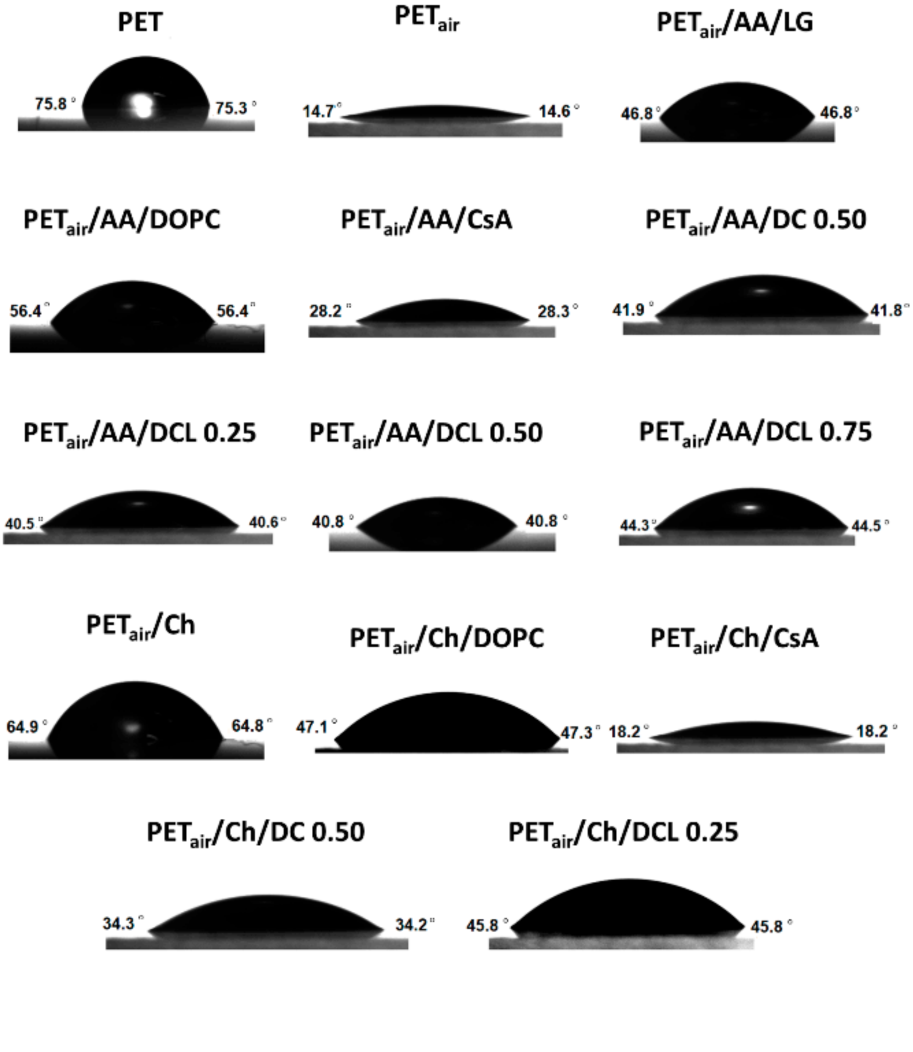

Figure S1. Water contact angle images for the indicated LB films deposited on PET_{air}.

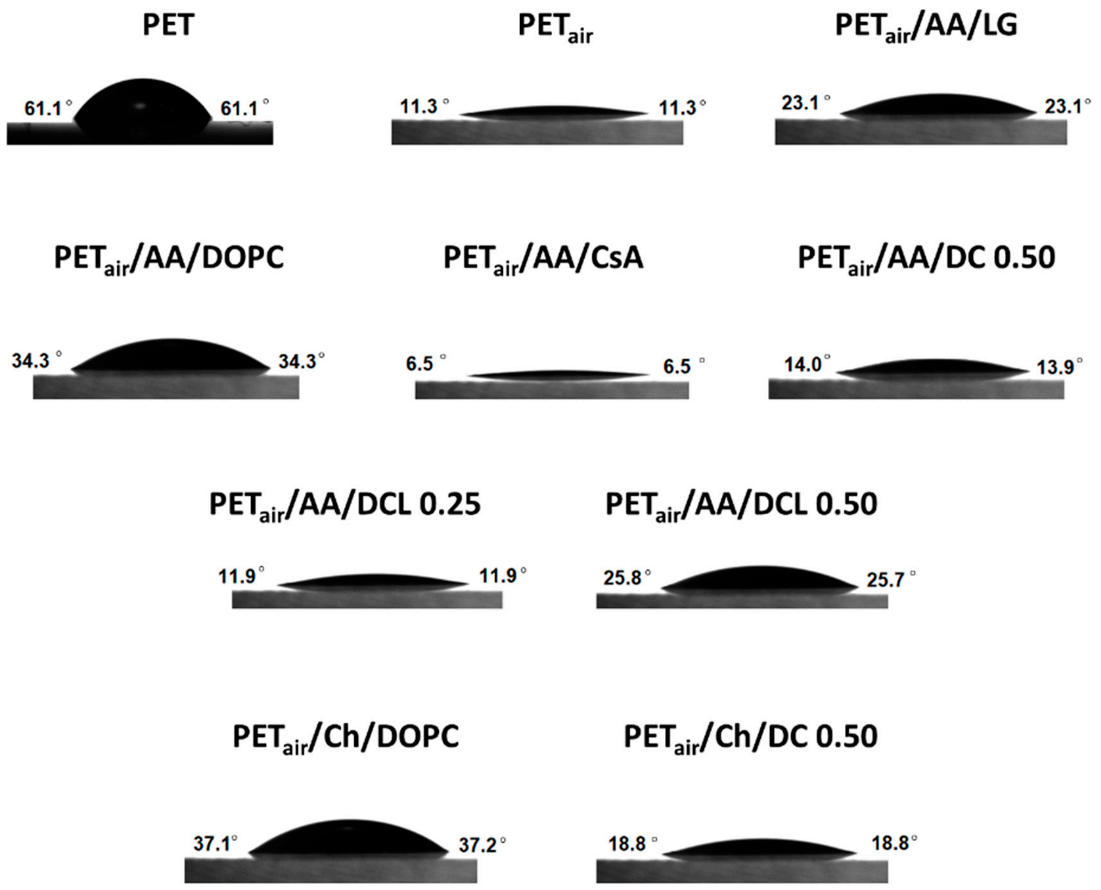

Figure S2. Formamide contact angle images for the indicated LB films deposited on PET_{air}.
